# Modulation of Septo-Hippocampal Neural Responses in Anesthetized and Behaving Rats by Septal AMPA Receptor Mechanisms

**DOI:** 10.3389/fncir.2021.663633

**Published:** 2021-06-04

**Authors:** Khairunisa Mohamad Ibrahim, Mohammed Zacky Ariffin, Sanjay Khanna

**Affiliations:** ^1^Department of Physiology, Yong Loo Lin School of Medicine, National University of Singapore, Singapore, Singapore; ^2^Neurobiology Programme, Life Sciences Institute, National University of Singapore, Singapore, Singapore; ^3^Healthy Longevity Translational Research Programme, Yong Loo Lin School of Medicine, National University of Singapore, Singapore, Singapore

**Keywords:** medial septum, AMPA receptors, NBQX, sensorimotor, theta activity, nociception

## Abstract

This study explored the effects of septal glutamatergic transmission on septal-hippocampal theta activity *via* intraseptal microinjection of antagonist at AMPA receptors (AMPAR). The current results showed that microinjection of AMPAR antagonist, NBQX (2,3-dihydroxy-6-nitro-7-sulfamoyl-benzo[f]quinoxaline-2,3-dione, 20 μg/μl, 0.5 μl), evoked a decrease in the frequency of theta activity evoked by various means in anesthetized and behaving rat. Theta wave activity was induced on: (a) intraseptal microinjection of carbachol, an agonist at cholinergic receptors, (b) reticular stimulation, (c) exploration in novel open field (OF), and (d) hind paw (HP) injection of the algogen, formalin. The effect on frequency in the formalin test was observed in an early period on injection of formalin, which was novel to the animal, but not in the later more sustained phase of the formalin test. The effect of NBQX, being seen in both anesthetized and behaving animals, suggests that the modulation of theta wave frequency, including in novelty, is a function of AMPAR in MS. The effect of the antagonist on theta power was less apparent, being observed only in anesthetized animals. In addition to theta power and frequency, intraseptal NBQX also attenuated suppression of CA1 population spike (PS) induced by intraseptal carbachol, thus suggesting that septal glutamate neurotransmission is involved in the spectrum of MS-mediated network responses. Indeed, in the context of behavior, formalin injection induced an increase in the level of septal glutamate, while NBQX attenuated nociceptive behaviors. Notably, MS is involved in the modulation of formalin nociception. These findings suggest that AMPA receptors are a key modulator of septal physiological function.

## Introduction

The medial septal region (MS) is implicated in various aspects of behavior such as affect-motivation, cognition, behavioral arousal, and sensorimotor integration (Winson, 1978; Gray, 1982; Bland, 1986, 2009; Givens and Olton, 1990; Nagahara and McGaugh, 1992; Gray and McNaughton, 2000; Bland and Oddie, 2001; Ma et al., 2002; McNaughton and Corr, 2004; McNaughton et al., 2006; Shin et al., 2009; Ang et al., 2015, 2017; Wang et al., 2015). Additionally, our laboratory has also shown that MS is a nodal region in mediating persistent unprovoked nociception and peripheral hypersensitivity (PH) to external stimulus following peripheral injury in rodents (Khanna and Sinclair, 1992; Khanna, 1997; Zheng and Khanna, 2001; Tai et al., 2006; Lee et al., 2011; Ang et al., 2015; Ariffin et al., 2018). Functionally, the MS regulates hippocampal theta wave activity which is a 4–12 Hz extracellular sinusoidal waveform that provides the basis for temporal organization of information in the hippocampus (Leung, 1984; Bland, 1986; Buzsáki, 2002). For example, an impairment of spatial memory correlates with the extent of decrease in hippocampal theta upon septal lesion, while restoring hippocampal theta rhythmicity with intracerebral stimulation rescued learning impairment caused by septal inactivation (Winson, 1978; McNaughton et al., 2006). Theta synchronization is also observed in parallel with voluntary behaviors and its amplitude and frequency may vary with moment-to-moment shifts in behavior (McFarland et al., 1975; Bland et al., 2006; Vandecasteele et al., 2014; Bender et al., 2015; Barth et al., 2018).

The septal glutamatergic neuropil and intraseptal glutamatergic transmission have been proposed to affect a wide range of septo-hippocampal functions, including theta activation, sensorimotor behaviors, PH, and cognition (Elvander-Tottie et al., 2006; Fuhrmann et al., 2015; Ariffin et al., 2018). Although stimulation of intraseptal afferent in theta range induced AMPA currents, the role of these receptors in theta mediation still remains unclear (Armstrong and MacVicar, 2001; Leung and Shen, 2004; Bland et al., 2007). While there is evidence of AMPAR-mediated nociceptive process in experimental neuropathic pain (Ariffin et al., 2018), there is a lack of information on its role in acute nociception within the septo-hippocampal network.

Therefore, in this study, we first investigated whether septal AMPAR mechanisms are involved in septo-hippocampal network responses in anesthetized animals. The septo-hippocampal activation was induced indirectly by reticular stimulation, or directly by intraseptal microinjection of AMPA and carbachol. Both induced a robust hippocampal theta activation and suppression of pyramidal cell somatic excitation of hippocampal CA1. These mimic the processing observed in the formalin test and during behavioral arousal (Khanna, 1997; Zheng and Khanna, 2001). We then used the AMPAR antagonist NBQX to examine the effect of the antagonist on theta activation and behavior induced by hind paw (HP) injection of the algogen, formalin, and on exposure to a novel open field (OF). The formalin injection induces animal ambulation and theta activation that parallels changes in nociceptive behaviors. Thus, this model is useful to evaluate septal glutamatergic modulation of sensory-related behaviors, sensorimotor integration, and related theta activation. Additionally, the release of glutamate in the MS during open field exploration and formalin-induced behaviors was also explored using the amperometric technique.

## Material and Methods

### Animals

Seven-week-old adult male Sprague–Dawley rats (270–330 g at the time of surgery) were used. The experiments followed the Ethical Guidelines of the International Association for the Study of Pain (Zimmermann, 1983) and were approved by the Institutional Animal Care and Use Committee (IACUC) at the National University of Singapore (NUS).

#### Surgery and Electrode Placements in Anesthetized Animals

The animals were anesthetized with urethane (1 g/kg, i.p.; Sigma, USA), mounted onto a stereotaxic frame, and prepared for electrophysiological recording from left hippocampus field CA1 (AP 4.0 mm, ML 2.5 mm, DV 2.0–3.0 mm from the cortical surface; Paxinos and Watson, 2014) by following the procedure as previously described (Jiang and Khanna, 2004, 2006). Urethane was prepared as 50% w/v solution made by dissolving the crystals in heated saline. The signal recorded by a carbon-fiber glass microelectrode was amplified 500× and split into two to record field activity (1–100 Hz) and population spike (PS; 1–1,000 Hz). Recordings were taken from the pyramidal cells layer which was determined by the presence of characteristic complex spike cell activity. CA1 PS was generated on stimulation of ipsilateral field CA3 (AP 3.0 mm, ML 2.0 mm, AV 4.0 mm from the cortical surface; 0.1 Hz, 0.01 ms pulse duration) with a stainless steel concentric bipolar electrode (Model NE-100, David Kopf, USA). The CA3 stimulation intensity was adjusted to evoke PS at either 75% or 25% of maximal PS amplitude (annotated as 75% PS or 25% PS respectively) under large irregular field activity (LIA).

#### Reticular Stimulation in Anesthetized Animals

In these animals, hippocampal theta wave activity was generated by stimulating the rostral pontine oralis nucleus (RPO; AP 8.0 mm, ML 0.8 mm, DV 9.0 mm from the cortical surface) of the reticular formation, ipsilateral to the recording site. The RPO was stimulated every 10 s for a duration of 2.56 s (100 Hz train, 0.01 ms pulse duration) using a stainless steel concentric bipolar electrode (Model NE-100, David Kopf, USA). The CA3 was also stimulated 10 ms from the end of RPO stimulation. The RPO stimulation was adjusted to evoke theta activity at ~ 4–5 Hz and suppression of the CA1 PS (Jiang and Khanna, 2004).

#### Drug Microinjection in Anesthetized Animals

The drug(s) were microinjected either using a single 33G microinjection stainless steel needle/cannula coupled to an Exmire syringe (Ito Corporation, Japan) or *via* a double-barreled silicon cannula assembly made by fusing two 33G silicon cannula (WPI, USA; Ariffin et al., 2010). The microinjection cannula was directed at a 14° angle to the vertical to the MS region. The two cannula barrels were oriented along the rostral-caudal axis and were used for pharmacological investigations that involved the microinjection of an agonist and an antagonist into the MS. The single 33G needle was used to microinject glutamate antagonist in experiments involving intracerebral stimulation.

#### Surgery and Implantation of Cannula and Electrodes for Investigations in Behaving Animals

The procedure has been described previously (Lee et al., 2011; Ang et al., 2017; Ariffin et al., 2018). Briefly, rats were anesthetized with an isoflurane-oxygen mixture (5% for induction and 2% for maintenance) and mounted onto a stereotaxic frame with the surface of the skull in a horizontal plane. A single 26G microinjection stainless steel guide cannula (Plastic One) was implanted into the MS (AP 0.5 mm, ML 0.0 mm, DV 5.4 mm from the skull surface) and subsequently secured to the skull with stainless screws and dental cement (Shofu Inc., Kyoto, Japan). In most animals, hippocampal depth recording electrodes were implanted along with a septal microinjection cannula. This was to investigate the effect of drug microinjection on hippocampal field activity accompanying the behavioral responses. The electrodes were constructed by twisting a pair of stainless steel wires together (A-M system, Carlsborg, WA, USA) and implanted into the dorsal CA1 (Lee et al., 2011; Ang et al., 2015). The two tips of the electrode that were lowered into the brain were spaced 0.5–1 mm apart in the vertical direction. Separate gold-plated male connector pins (A–M Systems, USA) were soldered onto the free end of the wires. The male connector pins at the free end of all the electrodes, including a ground screw electrode, were pushed into a 9-pin ABS plug (Ginder Scientific, Nepean, ON, Canada) which was then secured onto the skull with dental cement (Shofu Inc., Kyoto, Japan).

For electrochemical analysis of septal extracellular glutamate levels, a guide cannula was directed towards the MS (AP 0.5 mm, ML 0.0 mm, DV 4.7 mm from the skull surface). The guide cannula was lowered at a more superficial depth as the biosensor protrudes out by 2.3 mm from the tip of the guide cannula. A small amount of dental cement was applied to stabilize the guide cannula. A plastic receptacle, which later held the wireless potentiostat to facilitate wireless recording, was also secured to the skull.

#### Microinjection in Behaving Animal

The procedure has been described previously (Lee et al., 2011; Ang et al., 2015; Ariffin et al., 2018). Briefly, the animal was restrained lightly while a stainless-steel internal cannula (33G, Plastics One, USA) was lowered through the guide cannula to microinject drug solutions. The experimenter was blind to the drug being administered. The drug was administered over 30 s. The internal cannula was left *in situ* for at least 1 min after drug administration to facilitate diffusion and minimize backflow of the drug. To test that the microinjection assembly system was functioning properly, some solution was pushed out before the insertion and upon removal of the internal cannula after drug microinjection.

#### Open Field and Hippocampal Field Activity Recording

Hippocampal field activity during exploratory behavior was recorded *via* a thin flexible wire screwed on to the animal’s head stage at one end and connected to an amplifier (500× amplification, band-pass filtered at 1–100 Hz, Grass amplifier, Astromed Inc, USA) at the other end *via* a commutator. Before the start of novel open field observation, baseline hippocampal field activity was recorded during exploration in a familiar environment adjacent to the novel location. Exploratory behavior of the animal, which was defined as purposeful locomotion when the animal crossed the cage or reared on hind paws, was recorded for 10 min.

Subsequently, the animal was microinjected with NBQX or vehicle and transferred to a novel behavioral room for the open field experiment. The open field experiment was conducted as previously described (Ang et al., 2015). The animal was introduced into the novel chamber (43.2 cm length × 43.2 cm width × 30.5 cm height) from the lower right corner at 5 or 15 min after drug microinjection. The time of drug pre-treatment was varied to determine whether the greater availability of the antagonist in MS with shorter pre-treatment would affect the behavioral response over time. The ambulatory behavior was monitored for 20 min. The animal’s ambulatory behavior (ambulatory distance traveled and speed) was captured by the infrared beams and quantified digitally using the activity monitoring software (ActiMot, Med Associates Inc., USA).

#### Formalin Test

Animal behavior in the formalin test was recorded together with hippocampal field activity in experiments that involved intraseptal microinjection of NBQX (see below). The animal was habituated for at least an hour to the test cage (43.2 cm length × 21.7 cm width × 30.5 cm height) and recording cable for three consecutive days before the experiment. On the test day, the baseline hippocampal field activity was recorded during exploration before NBQX (20 μg/μl) or vehicle was microinjected into the MS. As with the open field test, the formalin test was performed 5 or 15 min after the microinjection of the drug into the MS. The animal was restrained lightly and formalin (1.25%, 0.1 ml) was injected into the plantar surface of the right hind paw using a 27G needle (Tai et al., 2006). The animal was immediately returned to the chamber and the hippocampal field activity, together with the nociceptive behaviors (flinching and licking of the injured paw) was recorded for 90 min, except for experiments involving amperometric recording of glutamate current. The animal’s ambulatory behavior was also recorded using the infrared sensors of the activity monitor (see above).

#### Amperometric Analysis Using Glutamate Biosensor

This experiment investigated the change in septal extracellular glutamate concentration in the formalin test using an enzyme (glutamate oxidase)-based, glutamate-selective biosensor (Pinnacle Technology Inc., USA). The glutamate-sensitive biosensor was connected to a wireless potentiostat and placed inside the receptacle secured previously during surgery. The potentiostat, in turn, signaled to the recording software (Sirenia^®^ software) *via* Bluetooth. Changes in glutamate concentration around the electrode were transduced into electrical current (nA per s).

The biosensors were calibrated pre- and post-experiment to test the functionality of the biosensor. The calibration protocol was recommended by the manufacturer (Pinnacle Technology Inc., USA) and was previously published by Wakabayashi and Kiyatkin (2012). The biosensor oxidation current was measured by increasing L-glutamate concentration *in vitro* in the buffer. The oxidation of the electrode arises from H_2_O_2_ that is produced because of the enzymatic reaction between L-glutamate and the enzyme associated with the biosensor. The glutamate concentration was increased in three consecutive steps by adding boluses of 40 μl of 5 mM L-glutamate solution (Sigma, USA) into the buffer (100 mM phosphate buffer saline, pH 7.4. 20 ml; VWR, USA) at 1 min intervals. Negative control involved the addition of ascorbic acid (an interference solution; 50 μl of 100 mM solution; Sigma, USA) to the testing buffer. At the end of calibration, 20 ml of phosphate buffer saline was added to halve the glutamate concentration in the test buffer to determine the biosensor’s ability to detect a fall in glutamate concentration. Separate pre- and post-experiment calibration tests were done to test the sensitivity of the biosensor by adding lower concentrations of L-glutamate (0.01–1.0 μM) into a fresh testing buffer.

During the experiment, the animal was habituated as described above. The biosensor was inserted into the MS *via* the implanted guide cannula the night before the formalin test after carrying out the pre-experiment calibration. On the test day, the animal was acclimatized to the environment before carrying out the formalin test. The test involved hind paw injection of saline (0.1 ml) and formalin (1.25%, 0.1 ml) into the left and the right hind paw, respectively. The injections were about 70 min apart. A baseline amperometric current was recorded for 10 min before each hind paw injection. The recording was continued for 60 and 90 min after saline and formalin injections, respectively. In addition, animal ambulatory behavior and speed were also recorded as described above. In the experiments described above, we preferred to administer the saline injection into the hind paw before performing the formalin test. Hind paw injection of formalin can sensitize neurons in CNS which may confound the response to hind paw saline. Further, although formalin was injected after saline injection, the biphasic pattern of behavioral response that is characteristic of formalin was still observed. Since saline *per se* did not affect glutamate levels we did not perform repeated saline injections.

At the end of the experiment, the animal was anesthetized, and the glutamate biosensor was carefully removed from its guide cannula for post-experiment calibration (see above) to determine the integrity of the biosensor during the experiment.

### Drugs

The following drugs were administered into either the MS or the lateral septum (LS) region of the brain: (a) 2,3-dihydroxy-6-nitro-7-sulfamoyl-benzo[f]quinoxaline-2,3-dione (NBQX disodium salt; 0.5 μl of 10 and 20 μg/μl solution; MW: 380.24 g/mol; 26.30 and 52.60 mM; Tocris, USA), which is a glutamate AMPA receptor antagonist, (b) α-amino-3-hydroxy-5-methyl-4-isoxazolepropionic acid (AMPA; 0.5 μl of 0.044 μg/μl; MW: 222.20 g/mol, 198 mM; Tocris, USA), an agonist at glutamate AMPAR and, (c) carbamylcholine chloride (carbachol, 0.5 μl of 0.156 μg/μl; MW: 182.696 g/mol; 0.854 mM; Sigma, USA), a cholinergic receptor agonist (Jiang and Khanna, 2006). The drugs were prepared in vehicle which has Alcian blue dye (0.5% w/v in saline, Sigma, USA). The vehicle was used for “control” microinjections.

### Histology

As previously reported (Lee et al., 2011), after perfusion and fixation with 5% v/v of formaldehyde (VWR, Germany), the brain was sectioned into 100-μm coronal sections using a vibratome (Leica VT 1200 Semi-Automatic Vibrating Blade Microtome, Leica Microsystems, Germany) and stained using 0.5% w/v Cresyl violet stain (Sigma, USA).

### Data Analysis

#### Electrophysiological Analysis

All electrophysiological data were collected and digitized using A/D converter (Power 141) and Spike2 program (Cambridge Electronic Design, UK). The hippocampal field activity and PS were digitized at 256 Hz and 10 kHz, respectively.

The data were analyzed as previously reported (Jiang and Khanna, 2004, 2006; Tai et al., 2006; Ariffin et al., 2010; Lee et al., 2011). The following parameters were analyzed: (a) change in the duration of theta wave activity (s/min or s/5 min), (b) change in fast Fourier transform (FFT) peak power in theta range in anesthetized (3–6 Hz), and behaving animal (4–12 Hz; i.e., FFT theta peak power), (c) change in FFT peak frequency in the theta range (i.e., FFT theta peak frequency), and (d) change in amplitude (mV) of the PS.

During analysis of theta wave activity, the field trace was digitally band-pass filtered at 1–40 Hz (FIR filter). Data were calculated using artifact-free field activity traces. Duration of theta was determined by calculating the total time for which theta was visually identified as a continuous sinusoidal oscillation of at least 1 s duration at frequencies of 3–6 Hz or 4–12 Hz in anesthetized and behaving animals, respectively. FFT analyses (frequency resolution of 0.5 Hz) were performed on theta wave activity (minimum of 2 s) recorded during the experiment. The FFT theta peak power of RPO- and pharmacological-induced theta wave activity was normalized to the average FFT theta peak power in the baseline (pre-microinjection) period and the power of spontaneous theta, respectively. For statistical analyses in behaving animals, the theta peak power recorded during open field and the formalin test was normalized against the peak theta power recorded during exploration in the familiar environment just before the start of the respective experiments.

The hippocampal theta activity recorded in the formalin test was further analyzed offline by dividing it into sensory behavior-related and residual theta activity. Sensory behavior-related theta was defined as theta wave activity observed in parallel with formalin-induced flinching or licking of the injured paw. Stretches of theta wave activity of at least 2 s duration were used for analysis. In an event where nociceptive behavior was brief, for example, only one flinch was observed, a 2 s of theta wave activity (1 s before and 1 s after the flinch) was taken for analysis. Residual theta activity reflects theta wave activity that was not concurrent with formalin-induced nociceptive behaviors and may include theta wave activity that parallels formalin-induced ambulation.

#### Amperometric Analysis

The data obtained was analyzed for the following parameters: (a) time course of normalized glutamate current (nA). The current was normalized to baseline current before hind paw injection of saline or formalin, (b) average change in concentration of glutamate (μM) at selected time points for experiments that met the biosensor sensitivity current threshold. Based on the post-experiment calibration curve, the lowest L-glutamate concentration that was detected by all the glutamate biosensors used in this experiment was 0.1 μM which was taken as the threshold concentration that induces a measurable current. The mean current evoked by 0.1 μM L-glutamate was 0.034 ± 0.011 nA, which was used as the biosensor current sensitivity threshold.

The glutamate currents were analyzed using a custom-made program in MATLAB (MathWorks, USA). The current values recorded from the glutamate and null biosensors implanted into MS were binned in 5 min blocks to improve the signal to noise. The signal from the null biosensor was subtracted from the signal recorded from the glutamate biosensor to remove any signal inherent with the biosensor. The post-experiment calibration curves obtained for all electrodes showed a positive linear relationship with glutamate concentration. Thus, a regression line was fitted for each electrode and the concentration of glutamate at the specific time point of interest was calculated using the equation: *x* = (*y* − *c*) / *m*, where *y* is the current and *x* is the glutamate concentration. The calibration constants *m* and *c* for each electrode were obtained from the post-experiment calibration concentration curve regression line.

#### Behavioral Analysis

An ambulatory episode was recorded when the animal’s movement exceeded four in-built infrared beams (i.e., box size of about 10 × 10 cm) in 500 ms. The square box was centered on the animal and the distance between the center of the starting square position to the center of the next square position is taken as ambulatory distance traveled. Ambulatory speed is the ratio of the ambulatory distance traveled by the animal continuously in 2 s or more to the time taken to cover the distance. Licking was measured as the duration for which the dorsal and/or plantar region of the injured paw was licked by the animal, while flinching was the number of shakes of the injured paw.

#### Statistical Analysis

Data are expressed as mean ± S.E.M. The data were analyzed using one of the following statistical tests (Prism 8, GraphPad Software Inc.): (a) two-way repeated measures (RM) ANOVA followed by Bonferroni *post hoc* test of a time course, to analyze the effect of treatment within each time point or the effect of time within each treatment, (b) one-way ANOVA/one-way RM ANOVA followed by Newman-Keuls *post hoc* test to compare multiple groups, and (c) one-way RM ANOVA followed by Newman-Keuls *post hoc* to analyze change in the time course of a parameter in an experiment involving a single treatment. Statistical significance was accepted at *P* ≤ 0.05. In an event where data showed an unequal variance in Bartlett’s test, the data were normalized by log transformation and re-analyzed.

## Results

Theta wave activity was recorded from the stratum radiatum of field CA1 during experiments in behaving animals. The onset and offset of animal nociceptive behavior, i.e., flinching and licking were marked onto Spike2 during the formalin test. To gauge the effect of NBQX treatment on neural activities, experiments were performed in both anesthetized and awake animals.

### Effect of Intraseptal NBQX on RPO-Induced Neural Responses in Anesthetized Animals

**Figure 1:**
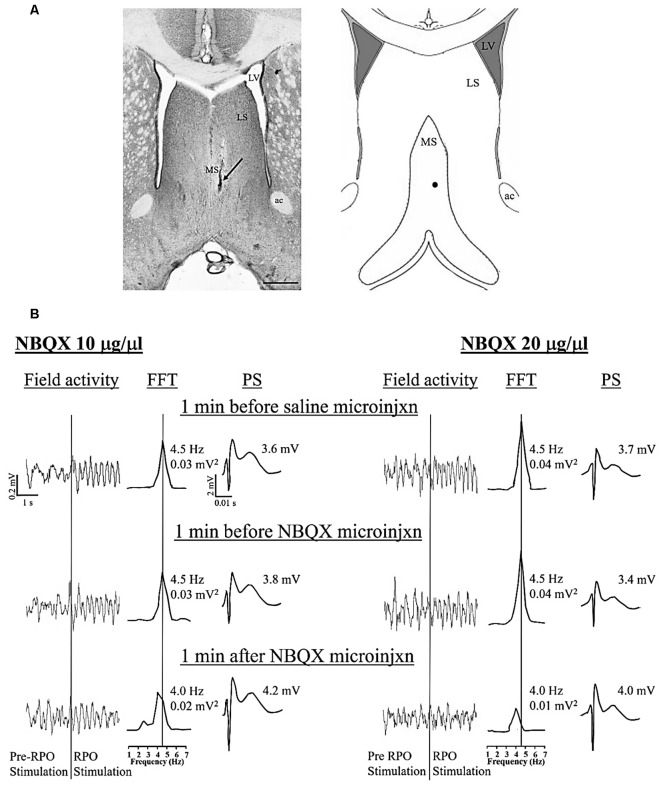
Dose-dependent effect of intraseptal microinjection of glutamate antagonist on reticularis pontis oralis (RPO) stimulation-induced hippocampal theta wave activity. **(A)** Digital representative image (left) of a microinjection site in the medial septum (MS) and the corresponding diagrammatic representation (right). The arrow (left) and the black dot (right) points to the Alcian blue dye, indicating the site of the microinjection. The scale bar on the digital micrograph represents 1.0 mm. The diagram on right is adapted from Paxinos and Watson (2014). **(B)** Representative electrophysiological responses recorded in the pyramidal cell layer of the hippocampus. The electrophysiological responses were taken from animals that received microinjection of 2,3-Dioxo-6-nitro-1,2,3,4-tetrahydrobenzo[f]quinoxaline-7-sulfonamide (NBQX, an AMPAR antagonist; 0.5 μl) at a dose of 10 μg/μl (left) or 20 μg/μl (right). The effect of each dose is illustrated on RPO stimulation-induced change in both hippocampal field activity (left) and the CA1 population spike (PS; right). The middle traces are fast Fourier transform (FFT; frequency resolution of 0.5 Hz) of the 2 s of RPO stimulation-induced field activity in the traces on the left of the FFT. RPO was stimulated every 10 s (0.1 Hz) for 2.56 s using a 100 Hz train (pulse width of 0.01 ms). FFT theta peak power, expressed as mV^2^ and normalized to the standard calibration unit, and the FFT theta peak frequency are indicated beside the FFT. The lines from the FFT theta trace at the top to the scale underneath indicate the FFT peak frequency corresponding to the FFT theta peak power. The PS was evoked on CA3 stimulation (0.1 Hz, 0.01 s pulse duration). The stimulation intensity was adjusted to evoke PS of about 75% of the maximal amplitude under conditions of large irregular field. The CA3 stimulation was timed to occur 10 ms after RPO stimulation. The amplitude of PS shown on the right of each PS trace reflects the average PS in 1 min block. During the experiment, vehicle (saline, 0.5 μl) was microinjected 10 min after the onset of RPO stimulation followed 10 min later by the microinjection of one of the two doses of NBQX. The electrophysiological responses illustrated in **(B)** were selected at three different time points as shown in the figure. Notice the reduction in both FFT theta peak power and frequency on microinjection of NBQX 20 μg/μl. Abbreviations: ac, anterior commissure; LS, lateral septum; LV, lateral ventricle; MS, medial septum; microinjxn, microinjection.

**Figure 2:**
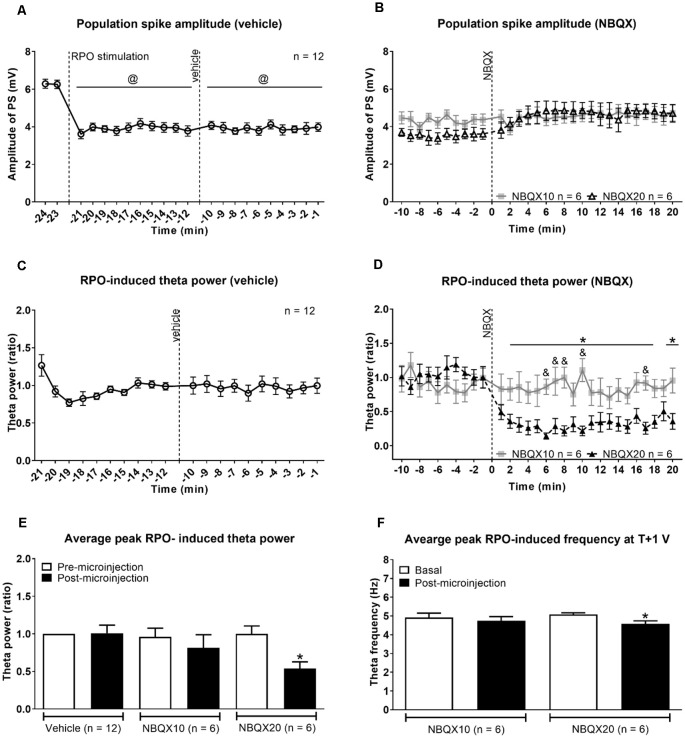
Intraseptal microinjection of NBQX affects power and frequency of reticular stimulation-induced hippocampal theta wave activity in a dose-dependent fashion. **(A,B)** Time course of effect of intraseptal microinjection of vehicle and NBQX on the reticular stimulation-induced suppression of the CA1 population spike (PS) and **(C,D)** the power of the concomitant theta wave activity. The RPO was stimulated at an intensity that evokes 4–5 Hz theta wave activity (labeled as ‘T’ V). The stimulation was initiated at the time point shown in **(A)** and was subsequently maintained throughout the recording except for intermittent periods when T + 1 V stimulation was applied. The plot in **(A)** corresponds to (a) 2 min before the onset of RPO stimulation (−24 min and −23 min), (b) 10 min following the onset of RPO stimulation, and before microinjection of vehicle (−22 min to −12 min), and (c) 10 min following vehicle microinjection (−11 min to −1 min). NBQX (10 or 20 μg/μl, 0.5 μl) was microinjected 10 min after vehicle microinjection **(A)**. This corresponds to 0 min in the plot **(B)**. The time course in **(B)** reflects 10 min before and 20 min after NBQX microinjection. Panels **(C,D)** are built as in **(A,B)** except power is not illustrated for the period before RPO stimulation since theta wave activity was not observed in this period. Furthermore, theta wave activity was lost around ~30 s to 1 min in four of six experiments following microinjection NBQX 20 μg/μl. In absence of overt theta, the peak FFT power in the theta range was used for drawing the plots. The FFT parameters and PS were computed in 1 min block. Power was expressed as a ratio of the average power in the 10 min preceding microinjection of vehicle. **(C)** As the effect of vehicle on PS and theta parameters was similar in both NBQX experiments, the vehicle data were combined for the two groups (*n* = 12). Note that the higher dose of NBQX attenuated theta power **(D)** but not the suppression of PS **(B)**. **(E)** Histogram of FFT theta peak power (ratio) before and after microinjection of vehicle and NBQX. The histograms represent the average of RPO-induced theta peak power in two contiguous minutes before microinjection and the average of peak RPO-induced theta peak power in two contiguous minutes in the 5 min period after microinjection. **(F)** Histograms showing FFT theta peak frequency before and after microinjection of NBQX at T + 1 V. Since NBQX 20 μg/μl evoked a loss of theta on RPO stimulation at intensity ‘T’, higher stimulation intensity at T + 1 was applied at 20 min after NBQX treatment to evoke theta wave activity which was then used for analysis. The theta frequency observed at 20 min post-NBQX was compared with the corresponding theta frequency evoked at T + 1 stimulation applied before the onset of time course investigations (Basal). Data are mean ± S.E.M. Significant difference (*p* < 0.05): **(A)** @vs. corresponding PS amplitude at −24, −23 min, one-way repeated measures (RM) ANOVA followed by Newman-Keuls *post hoc* test. **(D)** Time course: *NBQX20 vs. corresponding −2, −1 min, and ^&^NBQX20 vs. NBQX10, two-way RM ANOVA followed by Bonferroni *post hoc* test. **(E,F)** Histograms: *vs. pre-NBQX20 or Basal, two-tailed paired *t*-test.

This experiment investigated the effect of intraseptal NBQX microinjection (0.5 μl of either 10 or 20 μg/μl) on RPO-induced hippocampal theta field activity and the suppression of CA1 PS (Figures 1, 2). It has been proposed that the brainstem is important for mediating changes in CA1 excitability that accompany theta activation during behavioral arousal. Furthermore, the RPO-induced theta wave activity and suppression of PS are mediated through the hypothalamic supramammillary nucleus (SuM) and involve the MS (Jiang and Khanna, 2004; Ariffin et al., 2010). Interestingly, the SuM projection to MS is partly glutamatergic in nature (Leranth and Kiss, 1996). Thus, this experiment explored the glutamatergic nature of RPO stimulation-induced theta activation and suppression.

The protocol used was adapted from Jiang and Khanna (2004), where the time course of drug effect was monitored on hippocampal responses evoked with stimulation of RPO at an intensity threshold that evoked basal theta activity of ~ 4–5 Hz (T V or T volt). The experiment was designed such that the vehicle was microinjected first followed by NBQX (Figures 1, 2).

In addition, RPO was also stimulated at T + 1 V before the onset of time course investigations (Basal; Supplementary Figure 1) and at 20 min after NBQX microinjection (Figure 2F) to examine the effect of drug treatment on supra-threshold stimulation-induced responses. The suprathreshold stimulation was factored into the protocol to evoke theta wave activity in a situation when T V failed to elicit clear theta rhythm after application of NBQX. As illustrated below, this enabled verification of drug effect against an overt theta background. T + 1 V stimulation was not applied following vehicle microinjection as it was anticipated that vehicle will not affect the electrophysiological parameters. Indeed, as mentioned below and shown in Figure 2, vehicle pre-treatment does not influence theta parameters even at the lower intensity (T V) of RPO stimulation. Here it is notable that both the power and frequency of basal theta wave activity evoked on T + 1 V stimulation were higher than the corresponding values at T V stimulation (Supplementary Figure 1).

The onset of RPO stimulation induced a strong suppression of PS (Figure 2A). The average amplitude of PS in the 2 min before RPO stimulation was 6.27 ± 0.162 mV (*n* = 12), which significantly decreased to 3.80 ± 0.166 mV (*n* = 12; *t*_23_ = 12.38, *P* < 0.0001; two-tailed paired *t*-test) in the 2 min following RPO stimulation. The time course of PS suppression following the onset of RPO stimulation was sustained and unaffected by microinjection of vehicle (Figure 2A, *F*_5, 56_ = 17.93, *P* < 0.0001, one-way RM ANOVA). Although there seems to be an increase in PS amplitude across time post-NBQX20 microinjection, Bonferroni *post hoc* analysis did not indicate any significant difference compared to baseline values at −2 and −1 min (Figure 2B, time × treatment: *F*_29, 290_ = 1.702, *P* = 0.016, treatment: *F*_1, 10_ = 0.0311, *P* = 0.863, two-way RM ANOVA).

The administration of vehicle into the MS also did not have a significant effect on the time course of RPO-induced theta power (Figure 2C, *F*_11, 121_ = 0.315, *P* = 0.981, one-way RM ANOVA). Similarly, NBQX10 microinjection did not have a significant effect on the time course of RPO-induced theta power but the higher concentration of NBQX (NBQX20) induced a significant attenuation of FFT theta peak power along the time course compared to baseline (Figure 2D, time × treatment: *F*_29, 290_ = 4.896, *P* < 0.0001, treatment: *F*_1, 10_ = 6.533, *P* = 0.029, two-way RM ANOVA). It is important to note that theta wave activity was lost around ~30 s to 1 min in four of six experiments following microinjection of NBQX20. In absence of overt theta, the peak FFT peak power in the theta range was used for analysis of the time course.

The average peak RPO-induced theta power was also significantly reduced with NBQX20 but not on NBQX10 or vehicle microinjections (Figure 2E, *F*_5, 42_ = 2.907, *P* = 0.0242; one-way ANOVA followed by Newman-Keuls *post hoc* test). Note that the pre-microinjection theta power was similar across all groups.

As indicated above, theta wave activity evoked with T V stimulation was lost following microinjection of NBQX20. Since the lack of clear theta wave activity could mask the actual drug effects, we compared the effects of NBQX on theta parameters evoked with T + 1 V stimulation. In this context, theta parameters under T + 1 V stimulation recorded before the start of the experiment (Basal) and 20 min after microinjection (NBQX10 or NBQX20) were compared. Similar to T V stimulation, theta power at T + 1 V was also significantly attenuated by NBQX20 but not on NBQX10 microinjection (Supplementary Figure 1C, *F*_3, 20_ = 5.071, *P* = 0.0090; one-way ANOVA followed by Newman-Keuls *post hoc* test). Similarly, RPO theta frequency was attenuated by NBQX20 (Figure 2F; *F*_3, 20_ = 3.488, *P* = 0.0349; one-way ANOVA followed by Newman-Keuls *post hoc* test).

### Effect of Intraseptal NBQX on AMPA- and Carbachol-Induced Neural Responses in Anesthetized Animals

In this experiment, we examined if the selected dose of intraseptal NBQX (10 or 20 μg/μl, 0.5 μl) antagonizes the suppression of PS response (at 25% of maximal) and theta activation triggered by direct activation of MS neurons with intraseptal AMPA (0.044 μg/μl, 0.5 μl). A total of 25 animals were used in this experiment.

**Figure 3:**
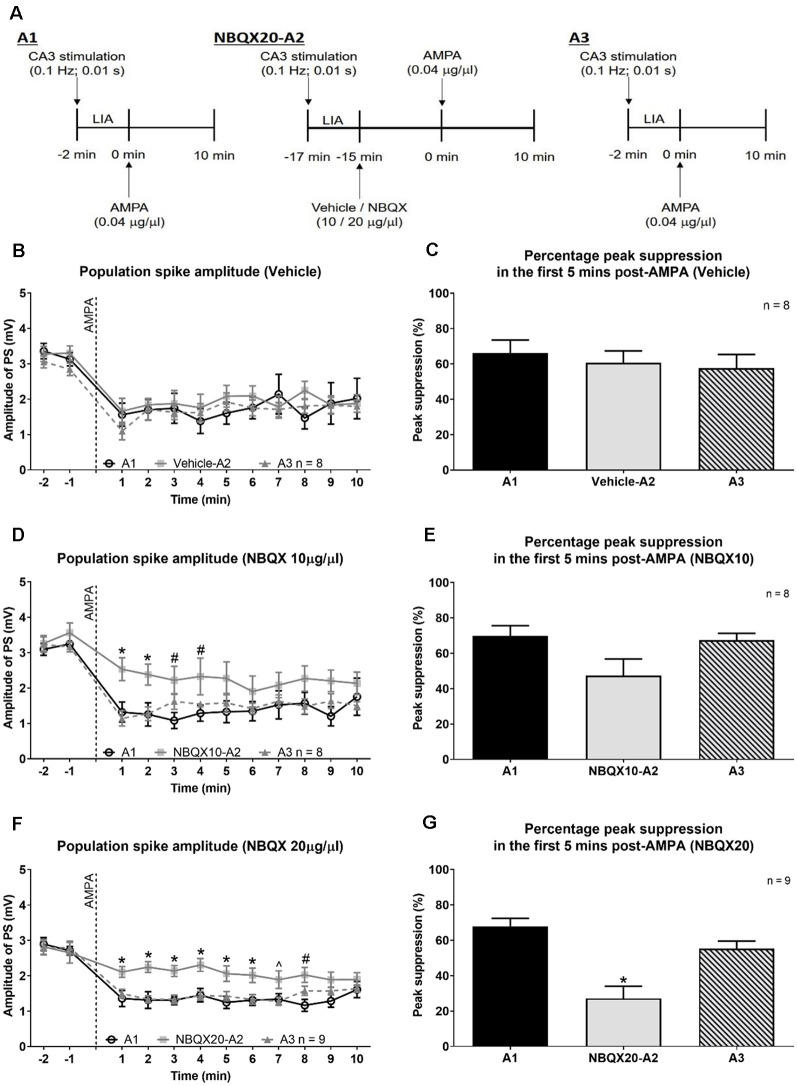
The highest dose of NBQX, AMPA receptor antagonist, antagonizes AMPA-induced suppression of CA1 population spike. **(A)** Experimental timeline to investigate the effect of NBQX (20 μg/μl, 0.5 μl) on AMPA (0.04 μg/μl, 0.5 μl)-induced suppression of dorsal hippocampus field CA1 population spike (PS). The CA1 PS was evoked with CA3 stimulation (0.1 Hz, 0.01 s pulse duration). The intensity of stimulation was adjusted to evoke control PS that was 25% maximal under large irregular field activity (LIA). AMPA was microinjected thrice, 1 h apart, into the MS (labeled A1, A2, and A3). The time of each AMPA microinjection was taken as 0 min. The effect of AMPA microinjection was continuously monitored for 10 min. A prefix, “NBQX20”, was added to A2 to indicate that NBQX was microinjected 15 min before the second AMPA microinjection (−15 min in **A**). The effect of microinjections on CA1 PS was analyzed in 1 min block. The time course of change is illustrated in **(B,D**, and **F)** wherein AMPA microinjection is indicated by the vertical line at time 0 min. The corresponding histograms **(C,E,** and **G)** show the percentage peak suppression induced in the first 5 min following AMPA microinjection. The percentage peak suppression was calculated from the average of the smallest PS amplitudes in two contiguous minutes within the first 5 min of AMPA microinjection expressed as a percentage of control PS amplitude. The control PS amplitude was the average amplitude in the 2 min before AMPA microinjection. Note that NBQX20 pre-treatment attenuated the suppression of CA1 PS induced by AMPA **(F,G)**. The effect of NBQX20 was persistent. In contrast, NBQX10 resulted in a brief block of AMPA-induced PS amplitude **(D)** with no significant decrease in percentage peak suppression **(E)**. Data are mean ± S.E.M. Significant difference (*p* < 0.05): **(D,F)** Time course: *NBQX20-A2 or NBQX10-A2 vs. A1 and A3, ^#^NBQX20-A2 or NBQ10-A2 vs. A1, ^∧^NBQX20-A2 vs. A3; two-way RM ANOVA followed by Bonferroni *post hoc* test. **(G)** Histograms: *vs. A1 and A3, one-way RM ANOVA followed by Newman-Keuls *post hoc* test.

During the experiment, AMPA was microinjected thrice (A1, A2, and A3), at least 1 h apart. NBQX or Vehicle was microinjected 15 min before A2 and denoted as NBQX10-A2, NBQX20-A2, or Vehicle-A2. CA1 PS was recorded for 2 min during the period of large irregular field activity (LIA) before each microinjection. The ability of NBQX to antagonize AMPA-induced responses was analyzed up to 10 min post-AMPA microinjection (Figure 3A).

Across all experimental groups the suppression of PS with first microinjection of AMPA (A1) was similar (Vehicle, 66.13 + 7.42% vs. NBQX10, 69.85 + 5.75% vs. NBQX20, 67.87 + 4.51%, Groups, *F*_2, 22_ = 0.0950, *P* = 0.9098; one-way ANOVA).

A two-way RM ANOVA revealed a lack of effect of Vehicle (Figure 3B; time × treatment: *F*_22, 231_ = 0.5102, *P* = 0.9681) and NBQX10 pre-treatment (Figure 3D; time × treatment: *F*_22, 231_ = 1.125, *P* = 0.3209) on the time course of AMPA-induced suppression of CA1 PS amplitude. In contrast, NBQX20 pre-treatment significantly attenuated the effect AMPA on PS amplitude (Figure 3F; time × treatment: *F*_22, 264_ = 1.873, *P* = 0.0117). Consistently, the AMPA-induced peak PS amplitude suppression was unaffected by Vehicle (Figure 3C; *F*_2, 14_ = 0.568, *P* = 0.589, one-way RM ANOVA) and NBQX10 pre-treatments (Figure 3E; *F*_2, 14_ = 3.408, *P* = 0.0623, one-way RM ANOVA). In contrast, NBQX20 pre-treatment attenuated the AMPA-induced peak PS amplitude suppression (Figure 3G; *F*_2, 16_ = 21.47, *P* < 0.0001, one-way RM ANOVA followed by Newman-Keuls *post hoc* test).

Robust theta activation was observed with A1 microinjections of AMPA across all experimental groups. The average duration of theta in the 10 min period after AMPA administration was similar (Vehicle, 36.42 + 2.29 s/min vs. NBQX10, 31.70 + 2.79 s/min vs. NBQX20, 38.57 + 2.95 s/min; *F*_2, 22_ = 1.672, *P* = 0.2109; one-way ANOVA followed by Newman-Keuls *post hoc* test).

**Figure 4:**
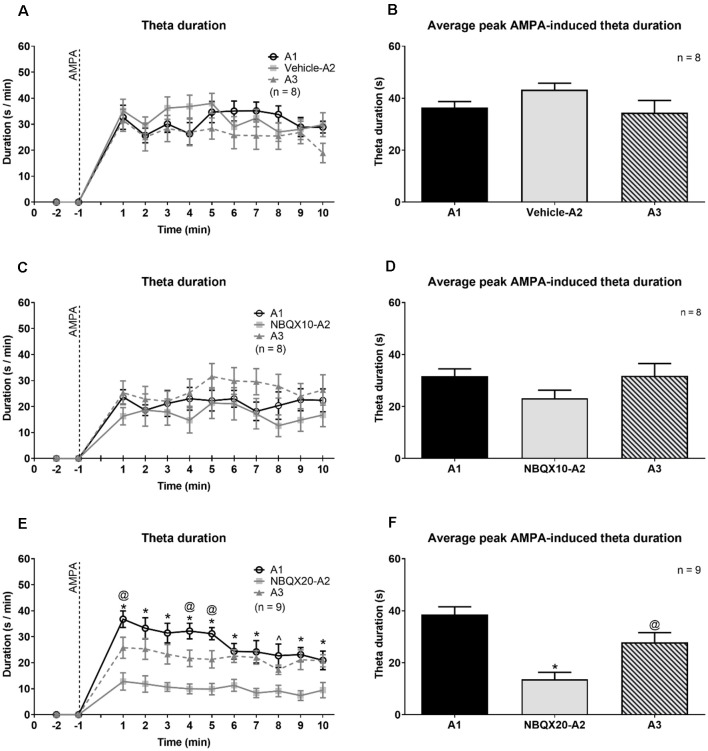
The highest dose of NBQX, AMPA receptor antagonist, attenuates the duration of theta wave activity induced on microinjection of AMPA. The figure is developed as explained in Figure 3. On left are time courses representating the effect of Vehicle **(A)**, NBQX10 **(C)** and NBQX20 **(E)** on the duration of AMPA-induced theta wave activity. The duration of theta was calculated as the sum of visible theta in a 1-min block. The histogram on right of the time courses **(B,D** and **F)** are the corresponding average peak AMPA-induced theta duration which was calculated as the average of the highest value of duration in two contiguous minutes in the first 5 min following microinjection of AMPA (left panel) and the histograms highlight the average highest value of that theta duration in two contiguous minutes in the first 5 min following microinjection of AMPA (right panel). Only the first 10 min, corresponding to the peak of AMPA effect following its’ microinjection, were analyzed. The protocol for drug microinjection is as explained in Figure 3. Note that NBQX (20 μg/μl), but not Vehicle or NBQX10, significantly antagonized AMPA-induced theta activation **(E,F)**. Data are mean ± S.E.M. Significant difference (*p* < 0.05): **(A,C,** and **E)** Time course: *NBQX20-A2 vs. A1 and A3, ^@^A3 vs A1, ^∧^NBQX20-A2 vs. A1; two-way RM ANOVA followed by Bonferroni *post hoc* test. **(B,D,** and **F)** Histograms: *vs. A1 and A3, ^@^vs. A1; one-way RM ANOVA followed by Newman-Keuls *post hoc* test.

NBQX20, but not NBQX10 and Vehicle pre-treatment, prevented the robust increase in duration of theta (Figures 4A,C,E). In this context, two-way RM ANOVA followed by Bonferroni *post hoc* test was used to compare duration of theta wave activity in the 10 min period post-AMPA microinjection. The analysis revealed that pre-treatment with Vehicle or NBQX10 had no significant effect on duration of AMPA induced theta activity (Figures 4A,C; Vehicle; time × treatment: *F*_22, 231_ = 0.8856, *P* = 0.6141; NBQX10; time × treatment: *F*_22, 231_ = 0.7376, *P* = 0.7981). However, NBQX20 significantly attenuated duration of AMPA-induced theta (Figure 4E; NBQX20, time × treatment: *F*_22, 264_ = 3.760, *P* < 0.0001). Consistently, the AMPA-induced average theta duration was attenuated by NBQX20 but not by other pre-treatments (Figures 4B,D,F; Vehicle, *F*_2, 14_ = 1.780, *P* = 0.2047; NBQX10, *F*_2, 14_ = 3.638, *P* = 0.0534; NBQX20, *F*_2, 16_ = 19.01, *P* < 0.0001; one-way RM ANOVA followed by Newman-Keuls *post hoc* test).

Interestingly, the frequency and power of remaining theta following NBQX20 pre-treatment (NBQX20-A2) was no different from A1 AMPA-induced theta. Further, neither Vehicle nor NBQX10 pre-treatment attenuated AMPA-induced theta power and frequency. The corresponding statistics (one-way RM ANOVA followed by Newman-Keuls *post hoc* test) are as follows: (a) Vehicle pre-treatment (Supplementary Figures 2A,B) - theta frequency: *F*_2, 14_ = 0.665, *P* = 0.529; theta power: *F*_2, 14_ = 0.185, *P* = 0.832, (b) NBQX10 pre-treatments (Supplementary Figures 2C,D) - theta frequency: *F*_2, 14_ = 2.668, *P* = 0.104; theta power: *F*_2, 14_ = 1.303, *P* = 0.303, and (c) NBQX20 pre-treatment (Supplementary Figures 2E,F) - theta frequency: *F*_2, 14_ = 1.632, *P* = 0.227; theta power: *F*_2, 14_ = 0.587, *P* = 0.568. The observation, especially the lack of effect of NBQX20 on residual theta frequency and power suggests that such low level of residual theta activity was NBQX insensitive.

We next examined whether NBQX sensitive mechanism mediates, at least in part, the suppression and theta activation to intraseptal carbachol (0.156 μg/μl, 0.5 μl), a widely used pharmacological means of septal activation. Indeed, administration of carbachol into the MS has been previously shown to induce both a robust suppression of CA1 PS and theta activation (Zheng and Khanna, 2001). Furthermore, septal neurons that are recruited for theta rhythmogenesis are modulated by both cholinergic and glutamatergic inputs (Hajszan et al., 2004; Manseau et al., 2005).

The effect of NBQX20 was tested against carbachol-induced PS amplitude suppression at 25% or 75% of maximal amplitude. Comparison of the peak effect within the first 5 min of carbachol administration showed that NBQX20 did not affect the carbachol-induced suppression of 25% PS amplitude (Supplementary Figure 3A, *F*_2, 5_ = 0.769 *P* = 0.489, one-way RM ANOVA). In contrast, suppression of 75% PS amplitude by carbachol was blocked by NBQX20 (Supplementary Figure 3B, *F*_2, 12_ = 14.50, *P* = 0.0006, one-way RM ANOVA followed by Newman-Keuls test).

Although the basal PS amplitudes were kept different, the spontaneous theta frequency and power of 25% and 75% PS groups were statistically similar (frequency: *T*_11_ = 0.593, *P* = 0.565; power: *T*_11_ = 1.671, *P* = 0.123; two-tail unpaired *t*-test). In addition, theta activation induced by first carbachol microinjection (C1) in both groups was also comparable (duration: *T*_11_ = 0.381, *P* = 0.710; frequency: *T*_11_ = 1.419, *P* = 0.184; power: *T*_11_ = 0.499, *P* = 0.628; two tail unpaired *t*-test). Therefore, the data for the two groups were combined for theta analysis. NBQX20 had a significant effect on carbachol-induced theta, attenuating all theta parameters (Supplementary Figures 3C–E; duration: *F*_2, 24_ = 12.15, *P* = 0.0002; frequency: *F*_2, 24_ = 7.162, *P* = 0.0036; power: *F*_2, 24_ = 6.518, *P* = 0.0055; one-way RM ANOVA followed by Newman-Keuls *post hoc* test). It is also notable that the effect on theta frequency and power was sustained (Supplementary Figures 3D,E).

Interestingly, carbachol (C1, *n* = 13) induced a more robust theta than AMPA (A1, *n* = 33) in untreated animals. The average theta duration induced by carbachol was significantly higher compared to that induced by AMPA (C1: 37.32 + 1.961 s vs. A1: 27.65 + 0.577 s; *t*_18_ = 4.733, *P* = 0.0002; two-tail unpaired *t*-test). Similarly, carbachol induced a higher theta frequency (C1: 4.619 + 0.157 Hz vs. A1: 3.78 + 0.0158 Hz; *t*_18_ = 5.306, *P* < 0.0001; two-tail unpaired *t*-test) and theta power (normalized to spontaneous theta power; C1: 1.190 + 0.031 vs. A1: 1.057 + 0.025; *t*_18_ = 3.406, *P* = 0.0032; two-tail unpaired *t*-test) than AMPA. These observations suggest that experiments with carbachol microinjection provided a greater width than intraseptal AMPA to explore changes in theta activation. Whereas the theta frequency induced on microinjection of AMPA was closer to the threshold that defines theta in anesthetized animals. Potentially, therefore, in experiments that examined the effect of NBQX20 on intraseptal AMPA-induced theta, an attenuation of theta frequency by NBQX may lead to loss of theta wave activity thus masking the effect of the antagonist on AMPA-induced theta generation.

While we did not examine the effect of intraseptal Vehicle (saline) on carbachol-induced responses, it is notable that Vehicle pre-treatment does not affect AMPA-induced responses, which are less intense, especially theta activation, than those evoked by carbachol. Thus, we anticipate that intraseptal Vehicle will not affect carbachol-induced responses. Therefore, we viewed the “Vehicle” control group in the AMPA study as a surrogate to Vehicle control in the experiments with carbachol.

### Effect of Intraseptal Microinjection of Glutamate Antagonists on Behavior and Theta Activation in Novel Open Field

The previous experiments provided evidence that indirect and direct activation of the septo-hippocampal network *via* the septum occurs *via* an NBQX sensitive mechanism. In this experiment, we examined whether NBQX modulated hippocampal theta and animal behavior on exploration of the novel open field during which the septo-hippocampal network exhibits a robust activation. In separate experiments, Vehicle or NBQX (20 μg/μl) was microinjected into the MS 5 min or 15 min before exposure to the open field arena with the concurrent recording of field activity in the dorsal hippocampal field CA1 (Supplementary Figure 4). The time of NBQX pre-treatment was varied to determine whether the greater availability of the antagonist in MS with shorter pre-treatment would make a difference to the behavioral response over time.

For animals in the Vehicle 5 min and Vehicle 15 min pre-treatment groups, the ambulatory distance at 5 min (720.03 ± 120.17 vs. 520.03 + 68.35) and 20 min (199.1 ± 74.35 vs. 94.71 + 52.14) of the open field test was similar (two-tailed unpaired *t*-test; 5 min, *t*_10_ = 1.447, *P* = 0.1786; 20 min, *t*_10_ = 1.150, *P* = 0.2770). Likewise, the ambulatory speed, theta duration, FFT theta peak frequency, and FFT theta peak power was also comparable (5 min; *t*_10_ = 0.0879 at least, i.e., the least value seen among the different comparisons, *P* = 0.4178 at least; 20 min, *t*_10_ = 0.02768 at least, *P* = 0.2655 at least). Thus, Vehicle pre-treatment groups were grouped. Similarly, the corresponding parameters recorded in the open field were comparable between the NBQX 5 min or NBQX 15 min pre-treatment groups. Thus, these two groups were also combined.

**Figure 5:**
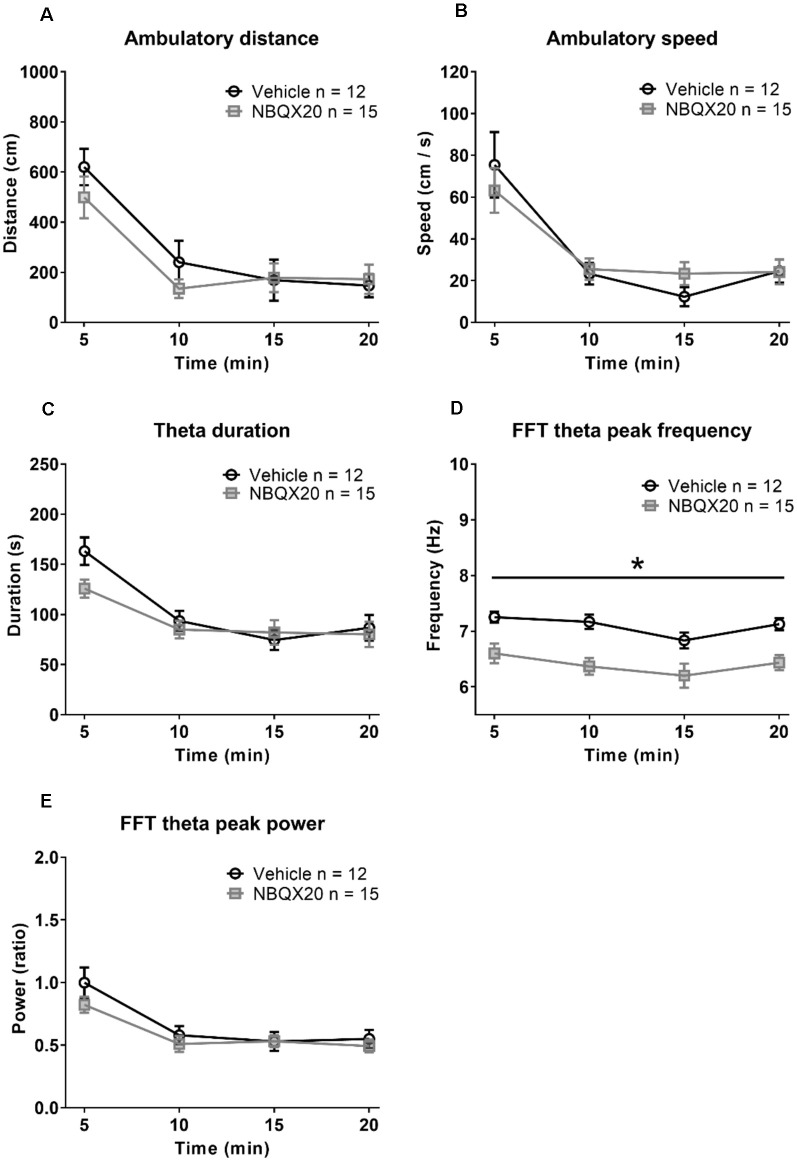
Intraseptal microinjection of NBQX 20 μg/μl attenuated theta wave frequency in novel open field (OF) with no effect on exploratory behavior. The figure illustrates the time courses of effects of intraseptal NBQX (20 μg/μl, 0.5 μl; ‘NBQX20’) on behavioral and electrophysiological parameters in a novel open field. The parameters measured included ambulatory distance **(A)**, ambulatory speed **(B)**, duration of theta wave activity **(C)**, FFT theta peak frequency **(D)**, and FFT theta peak power **(E)**. Vehicle or NBQX was microinjected either 5 or 15 min prior to the open field test. Since the effects of control microinjection of vehicle were very similar on the parameters, irrespective of time of microinjection, the data from the two control groups were combined. Likewise, for NBQX20. The theta duration was calculated as the sum of visible theta in 5 min block. The theta peak frequency (Hz) and theta peak power (peak-to-peak amplitude square in mV^2^ unit) represent the values obtained from FFT analysis. The theta power was expressed as a ratio of the pre-treatment theta power recorded during exploration in a familiar environment before the experiment. NBQX20 induced a sustained decrease in FFT theta peak frequency **(D)** without any effect on the duration of theta activity **(C)** and FFT theta peak power **(E)** over the 20 min of observation. The ambulatory distance **(A)** and velocity **(B)** remained were not affected by the antagonist. Data are mean ± S.E.M. Significant difference (*p* < 0.05): Time course: *NBQX20 vs. vehicle; two-way RM ANOVA followed by Bonferroni *post hoc* test.

The animals habituated with time resulting in decreasing exploratory behavior over time in both the Vehicle and NBQX20 pre-treatment groups (Figures 5A,B). Two-way RM ANOVA of the time courses of responses (Figures 5A–C,E) in open field showed that pre-treatment with NBQX20 did not affect ambulatory distance (time × treatment, *F*_3, 75_ = 1.209, *P* = 0.3125), ambulatory speed (time × treatment, *F*_3, 75_ = 0.7125, *P* = 0.5476), theta duration (time × treatment, *F*_3, 75_ = 1.924, *P* = 0.1329), and theta power (time × treatment, *F*_3, 75_ = 1.398, *P* = 0.2499). However, theta frequency was decreased with NBQX20 pre-treatment (Figure 5D; time × treatment, *F*_3, 75_ = 0.2378, *P* = 0.8691; treatment, *F*_1, 25_ = 15.25, *P* = 0.0006). Indeed, the average theta frequency in the 20 min test period was low in NBQX20 pre-treatment group vs. Vehicle pre-treatment group (6.400 + 0.1387 Hz vs. 7.094 + 0.096 Hz; *T*_6_ = 5.653, *P* = 0.0013, two-tail unpaired *t*-test).

### Investigation of the Change in Septal Glutamate Release in the Formalin Test

**Figure 6:**
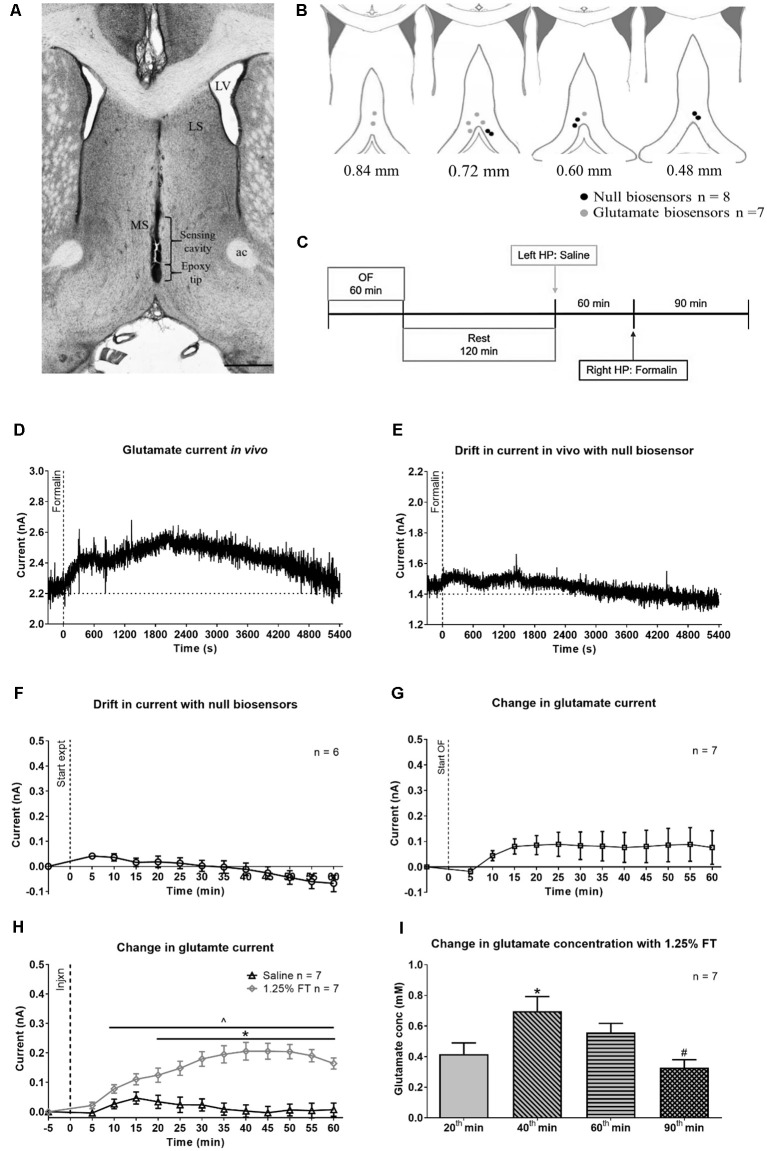
Change in glutamate current and concentration with hind paw (HP) injection of saline or 1.25% formalin. **(A)** Digital representative image of the track made by the biosensor in the midline MS. The deepest point of the track corresponds to the epoxy tip of the biosensor, which protects the sensing cavity of the biosensor during insertion through the implanted guide cannula. The sensing cavity is 1.0 mm long and is right above the epoxy tip. The scale bar on the image represents 1.0 mm. **(B)** Diagrammatic representation of the biosensor recording sites in the MS. The position of the epoxy tip of the null biosensor (black circles) and glutamate biosensors (gray circles) are shown. The diagram is adapted from Paxinos and Watson (2014). **(C)** Experimental timeline illustrating the recording of amperometric current in open field (OF) for 60 min followed by a 2 h rest in the home cage. The animal was sequentially injected with saline and 1.25% formalin into the left and the right hind paw (HP), respectively. The two hind paw injections were spaced 60 min apart. **(D)** Hind paw injection of 1.25% formalin (‘Formalin’ on the plot) into right hind paw induced a gradual increase in current recorded from glutamate biosensor. The increase was from a stable baseline before injection and peaked around 35 min (~2,100 s) post-formalin injection. Thereafter, the current gradually decreased towards the baseline at around 90 min post-formalin injection. **(E)** Variation in current recorded from null biosensor following 1.25% formalin injection into the right hind paw. **(F)** Time course showing the average drift in current for all null biosensors that were used in the study. **(G)** No significant change was observed with the current change during open field exploration. **(H)** The biosensor showed a sustained increase in current with injection (‘Injxn’ on the plot) of 1.25% formalin (1.25% FT), but not saline into hind paw injection. **(I)** Change in glutamate concentration (‘Glutamate conc’) at selected time points. All the seven glutamate biosensors used in the current study exceeded the estimated glutamate sensitivity threshold at the selected time points on injection of 1.25% formalin. Data are mean ± S.E.M. Significant differences (*p* < 0.05): Time course: *1.25% FT vs. saline, ^∧^1.25% FT vs. corresponding baseline, two-way RM ANOVA followed by Bonferroni *post hoc* test. Histogram: *vs. 20th and 90th min, ^#^vs. 60th min, one-way RM ANOVA followed by Newman-Keuls *post hoc* test.

The findings in the preceding section suggest that AMPA glutamatergic receptor mechanisms in MS modulate behavioral and electrophysiological indices in the novel open field. The septo-hippocampal network is known to mediate formalin nociception, the formalin test being a model of persistent inflammatory pain (Lee et al., 2011). However, the relative role of septal glutamatergic transmission in formalin-induced nociception, sensorimotor behaviors, and theta activation remains unclear. As a step towards exploring the role of septal glutamatergic receptor mechanisms in the formalin test, we used amperometric analysis to explore the change in glutamate levels within the MS in the formalin test. Glutamate levels were also examined in an open field test. The animals were habituated for three consecutive days to the experimental set up for the formalin test before the amperometric recordings on the day of the experiment. The recording electrodes were positioned in the MS (Figures 6A,B). A separate set of animals were implanted with null biosensors to record current drift that might have been due to the biosensor itself. All animals in this experiment follow the timeline as illustrated in Figure 6C.

Representative current traces recorded *in-vivo* from glutamate and null biosensor are shown in Figures 6D,E, respectively. The amperometric data was binned in blocks of 5 min. The average drift in the current recorded with the null biosensors (Figure 6F) was subtracted from the value recorded with the glutamate biosensors at the corresponding time block. The final extraction currents are shown in Figures 6G,H.

The change in glutamate current during exploration of an open field showed a trend towards an increase (Figure 6G). The increase, however, was marginally statistically insignificant as compared to its baseline (Figure 6G; *F*_12, 72_ = 1.882, *P* = 0.0511, one-way RM ANOVA). The total ambulatory distance covered by animals in 20 min of observation in the experiment was comparable to the total ambulation by Vehicle pre-treated animals exposed to a novel open field in Figure 5 (1056 ± 251.7 cm vs. 1248 ± 188.7 cm, *T*_20_ = 0.5537, *P* = 0.5859, two-tailed unpaired *t*-test).

In the formalin test, a gradual and sustained increase was observed with hind paw injection of formalin but not saline. A two-way RM ANOVA of the time course followed by Bonferroni *post hoc* test revealed a significant increase in glutamate current with formalin injection (Figure 6H, time × treatment: *F*_12, 144_ = 20.66, *P* < 0.0001, treatment: *F*_1, 12_ = 24.60, *P* = 0.0003). The increase in current was significant from the 20th min following injection of formalin. A Bonferroni *post hoc* test comparing the time points within each treatment group also revealed that the current post-formalin, but not saline injection was significantly higher than its corresponding baseline from 10th min onwards (Figure 6H). The estimated change in the glutamate concentration corresponding to the change in current recorded at selected 5 min time blocks is shown in Figure 6I. The increase was maximal at 40th min and declined subsequently (*F*_3, 18_ = 10.42, *P* = 0.0003; one-way RM ANOVA followed by Newman-Keuls *post hoc* test).

Here it is notable that nociceptive behaviors (i.e., flinching and licking of the injured paw) were not monitored in these experiments. Nonetheless, a biphasic increase in ambulatory distance and speed (distance: time × treatment, *F*_22, 198_ = 5.692, *P* < 0.0001, speed: time × treatment, *F*_22, 198_ = 4.147, *P* < 0.0001; Supplementary Figure 5) was observed on formalin injection which is typical of the formalin model (Lee et al., 2011). This biphasic response was not observed with hind paw injection of saline. Animals in the open field showed a peak in the ambulatory distance in the first 5 min followed by a gradual decline. It is interesting to note that formalin-induced ambulation (Supplementary Figure 5) declined towards baseline from 45th to 60th min, whereas the biosensor current remained elevated at a high level during that period (Figure 6H). Similarly, glutamate current continued to rise steadily during interphase with 1.25% FT where ambulatory and nociceptive behaviors are typically low. This suggests that the glutamate current reflects an accumulation of the neurotransmitter in the extracellular space over the course of the experiments rather than a moment-to-moment change in glutamatergic activity.

### Effect of Intraseptal Microinjection of Glutamate Antagonists on Formalin-Induced Nociceptive Behaviors

In these experiments, the effect of intraseptal microinjection of NBQX (20 μg/μl, 0.5 μl; denoted as NBQX20) was investigated on formalin-induced nociceptive behaviors and theta activation. In addition, NBQX was also microinjected into the lateral septum (LS) in a separate group of animals to investigate the site-specificity of the effect on formalin-induced nociception.

After habituation, formalin (1.25%, 0.1 ml) was injected into the right hind paw of the animals. Unlike the experiment above, the animals received only one injection as the saline injection was omitted in this study. NBQX20 or Vehicle was microinjected into either the MS or LS. The microinjection was performed at either 5 or 15 min before formalin injection. A two-tail unpaired *t*-test analysis showed that animals pre-treated with Vehicle at either 5 or 15 min before formalin had comparable responses in both phases 1 and 2 of the formalin test (Flinching: Phase 1, *t*_11_ = 0.8488, *P* = 0.4141, Phase 2, *t*_11_ = 1.381, *P* = 0.1946; Licking: Phase 1, *t*_11_ = 0.6632, *P* = 0.5208, Phase 2, *t*_11_ = 1.696, *P* = 0.1179). Therefore, the two groups were combined. Similarly, the responses in animals microinjected with NBQX into the LS at the two pre-treatment times were also similar and combined (Flinching: Phase 1, *t*_2_ = 0.8544, *P* = 0.4829, Phase 2, *t*_2_ = 0.2791, *P* = 0.8064; Licking: Phase 1, *t*_2_ = 0.5481, *P* = 0.6386, Phase 2, *t*_2_ = 0.5305, *P* = 0.6488).

**Figure 7:**
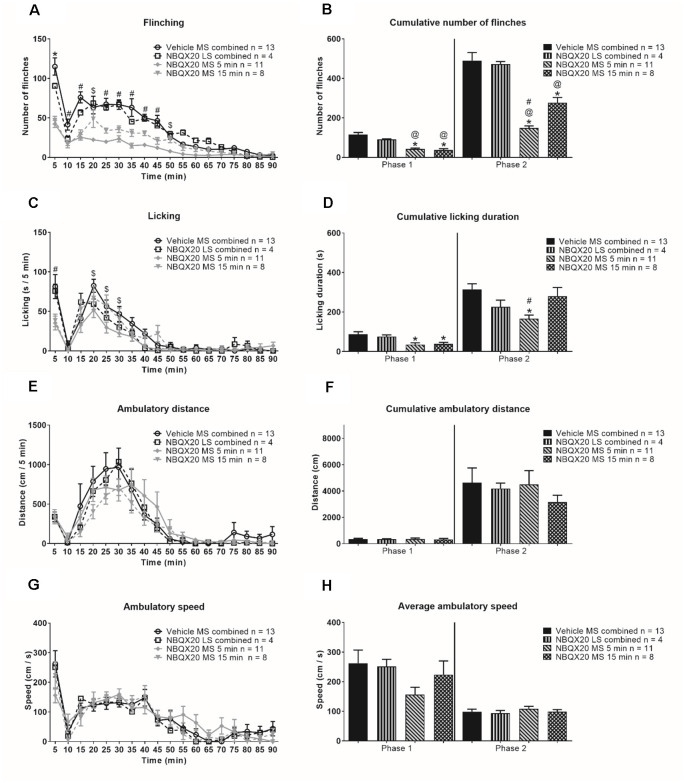
Intraseptal microinjection of NBQX selectively attenuates formalin-induced flinching and licking. The figure illustrates the time course effect of intraseptal NBQX (20 μg/μl, 0.5 μl; ‘NBQX20’) on formalin-induced behaviors. Formalin (1.25%, 0.1 ml) was microinjected into the right hind paw at either 5 or 15 min after microinjection of NBQX. The time of formalin injection was taken as 0 min. The nomenclature illustrated on the plots is as follows: treatment (vehicle or NBQX20), site of injection (MS or LS), and time of drug microinjection preceding formalin injection (5 min, 15 min, or ‘combined’ when 5- and 15-min groups are combined). The left panel shows the time course of the flinching **(A)**, licking **(C)**, ambulation **(E)**, and the ambulatory speed **(G)** calculated in 5-min block. The panels on the right are histograms showing the cumulative changes in behaviors in Phase 1 (0–5 min) and Phase 2 (11–60 min) of the formalin test **(B,D**, and** F)**. The histogram in **(H)** shows the average speed of ambulation in the two phases. The figure shows that NBQX20 microinjection in MS, but not LS, attenuated formalin-induced nociceptive behaviors with a stronger effect of microinjection of NBQX20 5 min before formalin injection. However, ambulation was not affected **(E)**. For clarity, only time points that showed a significant effect of NBQX compared to vehicle are demarcated on the graphs. Data are mean ± S.E.M. Significant differences (*p* < 0.05): Time course: *vehicle vs. NBQX20 MS 5 min, NBQX20 MS 15 min and NBQX20 LS combined, ^#^vehicle vs. NBQX20 MS 5 min and NBQX20 MS 15 min, ^$^vehicle vs. NBQX20 MS 5 min, two-way RM ANOVA followed by Bonferroni *post hoc* test. Histograms: *vs. Vehicle, ^@^vs. NBQX20 LS combined and ^#^vs. NBQX20 MS 15 min, one-way ANOVA followed by Newman-Keuls *post hoc* test.

Microinjection of NBQX20 into the MS but not LS, 5 or 15 min before hind paw injection of formalin, significantly attenuated formalin-induced flinching (Figure 7A, time × treatment: *F*_51, 544_ = 67.50, *P* < 0.0001, treatment: *F*_3, 32_ = 33.42, *P* < 0.0001, two-way RM ANOVA followed by Bonferroni *post hoc* test), in Phase 1 and Phase 2 of the formalin test (Figure 7B, Phase 1: Groups: *F*_3, 32_ = 20.22, *P* < 0.0001, and Phase 2: Groups: *F*_3, 32_ = 26.15, *P* < 0.0001, one-way ANOVA followed by Newman-Keul’s *post hoc* test). Interestingly, stronger suppression of flinching was observed in the NBQX20 MS 5 min group as compared to the NBQX20 MS 15 min group (Figure 7A). Similarly, a more prolonged decrease in formalin-induced licking was observed in the NBQX20 MS 5 min group (Figure 7C, time × treatment: *F*_51, 544_ = 1.894, *P* = 0.0003, treatment: *F*_3, 32_ = 3.923, *P* = 0.0172, two-way RM ANOVA followed by Bonferroni *post hoc* test). A decrease in formalin-induced licking behavior was observed in both Phase 1 and Phase 2 of the NBQX20 MS 5 min group, while a decrease was observed only in Phase 1 with the NBQX20 MS 15 min group (Figure 7D, Phase 1 Groups: *F*_3, 32_ = 5.190, *P* = 0.005; Phase 2 Groups: *F*_3, 32_ = 5.30, *P* = 0.004, one-way ANOVA followed by Newman-Keuls *post hoc* test).

NBQX microinjection into neither MS nor LS affected the time course of formalin-induced ambulatory distance and speed (Figures 7E and 7G, time × treatment: *F*_51, 544_ = 0.728, *P* = 0.921 and time × treatment: *F*_51, 544_ = 0.992, *P* = 0.493, respectively; two-way RM ANOVA). The two parameters were also similar in Phase 1 (ambulatory distance: Groups: *F*_3, 32_ = 0.024, *P* = 0.995; speed: Groups: *F*_3, 32_ = 1.532, *P* = 0.225) and Phase 2 (ambulatory distance: Groups: *F*_3, 32_ = 0.388, *P* = 0.762; speed: Groups: *F*_3, 32_ = 0.415, *P* = 0.744; one-way ANOVA) of the formalin test (Figures 7F and 7H).

In context of speed, the average speed in Phase 2 (68.88 + 12.56 cm/s, *n* = 9) of Vehicle pre-treated animals was less than Phase 1 (140.5 + 16.24 cm/s, *n* = 9; *P* = 0.010, two-tailed paired *t*-test). However, speed was registered in only two of the nine animals during interphase, the average speed in these animals being 90.86 cm/s (*n* = 2).

### Effect of Intraseptal Microinjection of NBQX on Formalin-Induced Hippocampal Theta Wave Activity

**Figure 8:**
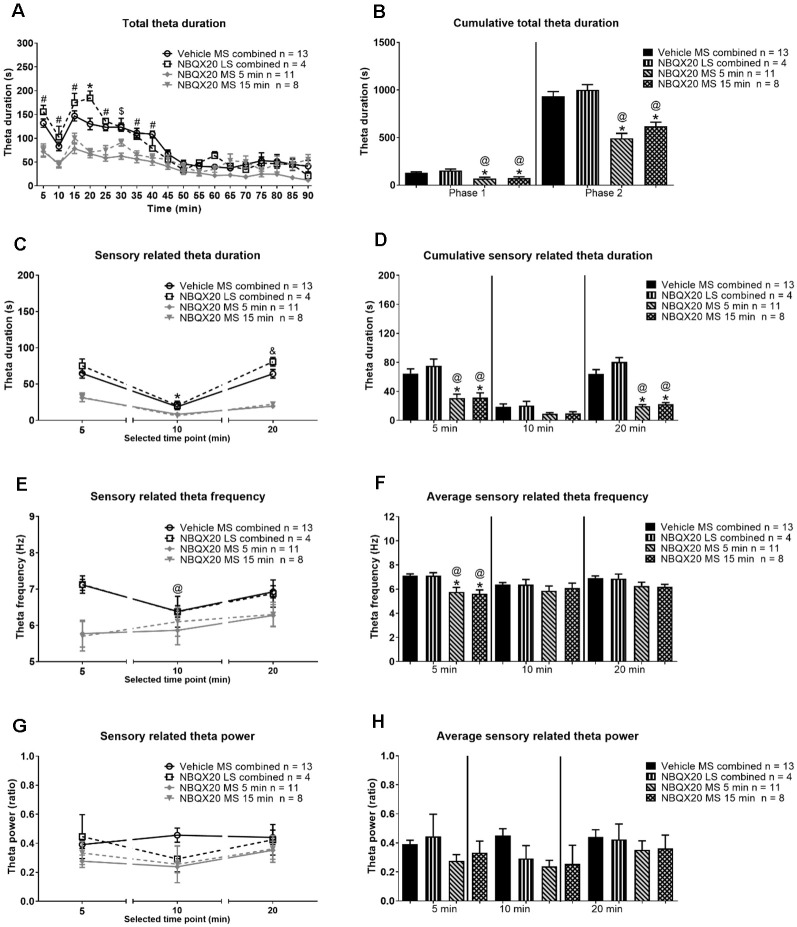
Intraseptal microinjection of NBQX attenuates formalin-induced hippocampal theta activity. The figure illustrates the effect of NBQX microinjection (20 μg/μl, 0.5 μl; ‘NBQX20’) on formalin-induced hippocampal theta wave activity. This figure is developed as in Figure 7. The left panels show the time course of the duration of total theta wave activity **(A)**, and the duration **(C)**, FFT theta peak frequency **(E)**, and FFT theta peak power **(G)** of sensory behavior-related theta wave activity at selected time points. The corresponding panels on the right are histogram representation of the parameters. The time points illustrated in **(C–H)** correspond to peaks of behavior and theta activation in Phase 1 (0–5 min) and Phase 2 (16–20 min) of the formalin test. The time point 10 min corresponds to the interphase (6–10 min; relatively quiescent phase) of the formalin test. The ‘Total theta duration’ reflects total theta activity which is inclusive of sensory-related theta and is presented in 5 min blocks **(A)** or the cumulative duration during Phase 1 (0–5 min) or Phase 2 (11–60 min) **(B)**. While ‘sensory theta’ is theta stretches that were observed in parallel with the formalin-induced flinching or licking behavior **(C–H)**. The duration and frequency of sensory theta **(C,E)** were lower at interphase suggesting a biphasic pattern. Further, microinjection of NBQX into MS, but not LS, affected all theta parameters except theta power. Data are mean ± S.E.M. Significant difference (*p* < 0.05): Time course: **(A)** *vehicle vs. NBQX20 MS 5 min, NBQX20 MS 15 min, and NBQX20 LS combined, ^#^vehicle vs. NBQX20 MS 5 min and NBQX20 MS 15 min, ^$^vehicle vs. NBQX20 MS 5 min; **(C,E)** *vs. 5 min for vehicle, NBQX MS 5 min, NBQX MS 15 min, and NBQX LS combined, and vs. 10 min for vehicle and NBQX LS combined, ^@^vs. 5 min for vehicle, two-way RM ANOVA followed by Bonferroni *post hoc* test. Histograms: *vs. Vehicle and ^@^vs. NBQX20 LS combined, one-way ANOVA followed by Newman-Keuls *post hoc* test.

The effect of intraseptal NBQX20 was also monitored on hippocampal theta wave activity recorded concomitantly with behavior during the formalin test. The hippocampal recording sites were in the stratum radiatum. Interestingly, the formalin-induced theta power was about half of that observed during exploration in a familiar environment (theta power ratio ~0.5, Figure 8G). In contrast, theta power induced during exploration in the novel open field, especially in the first 5 min was similar to that observed during exploration in a familiar environment (theta power ratio ~1.0, Figure 5E).

A robust biphasic increase in theta duration was observed in parallel with nociceptive behaviors following formalin hind paw injection (1.25%, 0.1 ml) in animals pre-treated with intraseptal vehicle (Figure 8A). A two-way RM ANOVA analysis showed a significant effect of intra-MS NBQX20 pre-treatment (5 or 15 min) on formalin-induced total theta duration as compared to Vehicle group (time × treatment: *F*_51, 544_ = 3.380, *P* < 0.0001, treatment: *F*_3, 32_ = 16.78, *P* < 0.0001). Microinjection into LS was not effective. Indeed, a phase analysis also showed that NBQX induced a significant decrease in cumulative total theta duration in both Phase 1 (Groups: *F*_3, 32_ = 10.78, *P* < 0.0001) and Phase 2 (Groups: *F*_3, 32_ = 21.30, *P* < 0.0001, Figure 8B) with microinjection into MS, but not LS.

The theta wave activity was separated into sensory related theta and residual theta activity. Sensory related theta activity is theta activity observed in parallel with formalin-induced flinching and licking of the injured hind paw. The residual theta wave activity reflects the activity recorded in periods marked by the absence of flinching and licking and includes theta wave activity which parallels formalin-induced ambulation. The theta activity was analyzed in blocks of 5 min that corresponded to the peaks of theta and nociceptive behaviors in Phase 1 (0–5 min or 5 min time point) and Phase 2 (16–20 min or 20 min time point), and the interphase which is the relatively quiescent period (6–10 min or 10 min time point) in the formalin test.

The duration of sensory related theta was biphasic with a significantly lower duration of theta at interphase as compared to Phase 1 and Phase 2 (Figure 8C, time × treatment: *F*_6, 64_ = 4.751, *P* = 0.0005, treatment: *F*_3, 32_ = 33.92, *P* < 0.0001, two-way RM ANOVA followed by Bonferroni *post hoc* test). NBQX20 significantly attenuated duration of sensory related theta at Phase 1 (Groups: *F*_3, 32_ = 9.873, *P* < 0.0001) and Phase 2 (Groups: *F*_3, 32_ = 32.98, *P* < 0.0001), but not interphase (Groups: *F*_3, 32_ = 2.36, *P* = 0.093, one-way ANOVA followed by Newman-Keuls *post hoc* test, Figure 8D), when microinjected into MS, but not LS.

**Figure 9:**
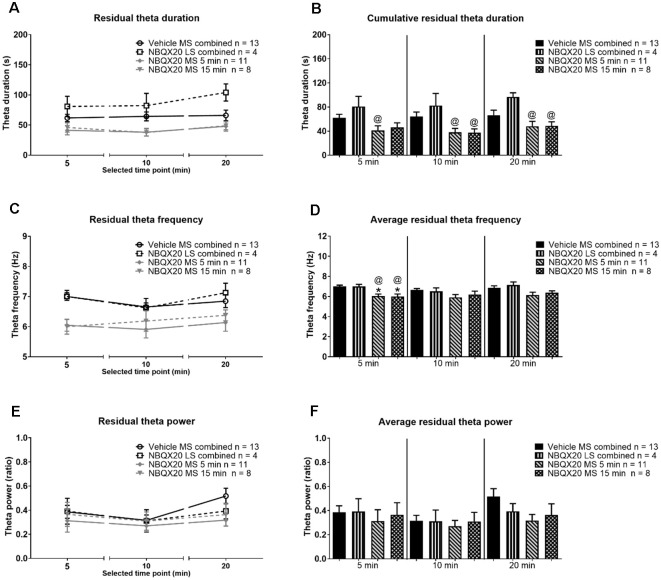
Intraseptal microinjection of NBQX affects residual theta. The figure illustrates the effect of NBQX microinjection (20 μg/μl, 0.5 μl; NBQX20) on residual theta parameters. The figure is developed as in Figure 8. The residual theta is theta activity observed at times other than those during formalin-induced flinching and licking. A decline in theta parameters at interphase, as compared to peaks of both Phase 1 (5 min) and Phase 2 (20 min), was not observed, indicating that residual theta is not biphasic **(A,B)**. Further, microinjection of NBQX into MS, but not LS attenuated the frequency of residual theta **(C,D)**. Microinjection of NBQX had no effect on the power of residual theta **(E,F)**. Data are mean ± S.E.M. Significant difference (*p* < 0.05): Histograms: *vs. Vehicle and ^@^vs. NBQX20 LS combined, one-way ANOVA followed by Newman-Keuls *post hoc* test.

In contrast, the duration of residual theta on microinjection of NBQX into MS was similar to control across different phases within the different group, although the duration tended to be slightly higher at 20 min block in animal injected with NBQX into LS (Figure 9A, time × treatment: *F*_6, 64_ = 0.376, *P* = 0.891, treatment: *F*_3, 32_ = 8.507, *P* = 0.0003, two-way RM ANOVA). Indeed, one-way ANOVA followed by Newman-Keuls *post hoc* test of the experimental groups indicated that the duration of residual theta wave activity observed with NBQX microinjection into MS was not different from the Vehicle microinjected group, although it was lower than the “NBQX20 LS combined” group (5 min: Groups: *F*_3, 32_ = 3.35, *P* = 0.031, 10 min: Groups: *F*_3, 32_ = 5.07, *P* = 0.006, and 20 min: Groups: *F*_3, 32_ = 4.01, *P* = 0.016, Figure 9B).

Interestingly, the FFT peak frequency of sensory theta showed a biphasic trend with a lower frequency at interphase in the control “Vehicle MS combined” and “NBQX20 LS combined” groups but not in the “NBQX20 MS 5 min” and “NBQX20 MS 15 min” groups (Figure 8E, time × treatment: *F*_6, 64_ = 0.194, *P* = 0.194, treatment: *F*_3, 32_ = 3.838, *P* = 0.019, two-way RM ANOVA followed by Bonferroni *post hoc* test). One-way ANOVA with Newman-Keuls *post hoc* analysis showed that microinjection of NBQX20 into MS significantly lowered theta frequency in Phase 1 (Groups: *F*_3, 32_ = 7.68, *P* = 0.0005) but not at interphase (Groups: *F*_3, 32_ = 0.588, *P* = 0.588) or Phase 2 (Groups: *F*_3, 32_ = 2.411, *P* = 0.085, Figure 8F).

Sensory related theta power was also invariant across different Phases within the different groups (Figure 8G, time × treatment: *F*_6, 64_ = 0.979, *P* = 0.447, treatment: *F*_3, 32_ = 1.656, *P* = 0.196, two-way RM ANOVA). One-way ANOVA analysis also showed that power was very similar across different groups in Phase 1 (Groups: *F*_3, 32_ = 1.365, *P* = 0.271), interphase (Groups: *F*_3, 32_ = 2.266, *P* = 0.099) and Phase 2 (Groups: *F*_3, 32_ = 0.44, *P* = 0.723, Figure 8H).

On the other hand, the FFT peak frequency of residual theta was also invariant across different phases within the different groups (Figure 9C, time × treatment: *F*_6, 64_ = 0.811, *P* = 0.566, treatment: *F*_3, 32_ = 4.870, *P* = 0.007, two-way RM ANOVA). Although, like sensory theta, NBQX microinjection into MS significantly attenuated residual theta frequency at Phase 1 (Groups: *F*_3, 32_ = 8.468, *P* = 0.0003) but not at interphase (Groups: *F*_3, 32_ = 1.945, *P* = 0.142), and Phase 2 (Groups: *F*_3, 32_ = 2.53, *P* = 0.074, one-way ANOVA followed by Newman-Keuls *post hoc* test, Figure 9D). It is notable that the theta frequency also tended to be lower at interphase and Phase 2 in NBQX20 MS groups as compared to Vehicle treated group.

Although there is a trend towards an increase in FFT peak power of residual theta wave activity at 20 min compared to 10 min in the Vehicle treated group, this was not observed for the other treatment groups (Figure 9E, time × treatment: *F*_6, 64_ = 0.916, *P* = 0.489, treatment: *F*_3, 32_ = 0.662, *P* = 0.582, two-way RM ANOVA followed by Bonferroni *post hoc* test). One-way ANOVA followed by Newman-Keuls *post hoc* analysis also showed that power was very similar among different groups in Phase 1 (Groups: *F*_3, 32_ = 0.189, *P* = 0.903), interphase (Groups: *F*_3, 32_ = 0.151, *P* = 0.929, and Phase 2 (Groups: *F*_3, 32_ = 0.191, *P* = 0.148, Figure 9F).

Collectively, the preceding findings highlight that the decrease in total theta duration with NBQX pre-treatment likely reflects the drug-induced decrease in sensory behavior-related theta, with decreases being in parallel with a decrease in nociceptive behavior. Whereas the duration of residual theta is unaffected which is consistent with the finding that NBQX pre-treatment does not attenuate non-sensory behaviors such as ambulation.

## Discussion

The current study provides novel evidence that MS glutamatergic transmission at AMPAR modulates the frequency of hippocampal theta wave activity and nociceptive behaviors. An effect of NBQX on nociceptive behaviors has been reported before (Ariffin et al., 2018). In the dose administered, NBQX antagonized intraseptal AMPA-induced suppression of CA1 PS and theta activation. Further, in the context of theta, microinjection of NBQX into the MS but not LS reduced frequency of theta induced by different physiological and pharmacological means, namely: (a) reticular stimulation, (b) direct stimulation of MS with intraseptal microinjection of the cholinergic agonist, carbachol, (c) animal exploration of the novel environment and (d) nociceptive behavior. Across the different preparations, NBQX reduced the frequency by ~0.5–1 Hz. The effect of NBQX, being seen in both anesthetized and behaving animals, suggests that modulation of the frequency of theta wave activity is a function of AMPAR in MS.

In behaving animals, the effect of NBQX on theta frequency was observed during the period of observation in the novel open field and in Phase 1 of the formalin test which corresponds to the 1st 5 min after hind paw injection of formalin. However, NBQX did not affect frequency in Phase 2 of the formalin test. Notably, there is an element of novelty in Phase 1 due to injection of formalin, while Phase 2 is not novel *per se* but, instead, reflects a continuation of the nociceptive experience that is driven after a hiatus (i.e., interphase) by changes triggered by initial damage on injection in Phase 1 (Tai et al., 2006). Collectively, the preceding suggests that intraseptal AMPAR plays a role in theta generation during novelty. In contrast, AMPAR antagonist do not affect theta during exploration in a familiar environment (Leung and Shen, 2004). Also, of note here is that co-administration of antagonists at AMPAR and NMDAR into MS of behaving animal attenuates theta activation on optogenetic stimulation of septal glutamatergic neurons (Fuhrmann et al., 2015). However, the role of AMPAR alone remained undefined in that study.

Within group comparisons suggest that the lack of NBQX effect on theta frequency at interphase and Phase 2 of the formalin test was not due to a nociceptive overdrive. Thus, NBQX did not affect theta frequency at interphase which is marked by an ebb in nociceptive behaviors. Further, NBQX decreased nociceptive behaviors, both in Phase 1 and at peak of Phase 2 but did not affect theta frequency in Phase 2. The nociceptive behaviors at peak of Phase 2 were like that in Phase 1 in NBQX treated animals. The effect of NBQX on theta frequency was also not due to sedation. In this context, the effect of NBQX on frequency was observed both in the novel open field and in the formalin test without an effect on ambulation. Consistently, the average speed of ambulation was also not affected by NBQX.

However, we did not monitor moment-to-moment acceleration. Such acceleration, instead of speed, relates to instantaneous changes in the frequency of theta (Kropff et al., 2021). In contrast to instantaneous frequency, we measured average frequency over a period. Nonetheless, the selective effect of NBQX on the average frequency in the first phase of the formalin is also unlikely due to differences in opportunity to accelerate in that period vs. other time-points of the formalin test. Thus, for example, both the ambulatory distance and speed increased from a low at interphase to a high at peak of Phase 2, a period in which the animal is likely to accelerate often. In the novel open field, NBQX evoked a similar decrease in average frequency across different time points, although both ambulatory speed and distance decreased from a high in the first 5 min. Therefore, the findings broadly suggest that NBQX modulated changes in average theta frequency under the condition of novelty, though this does not discount the possibility that it may also modulate instantaneous frequency during epochs of acceleration.

On the contrary, intraseptal NBQX failed to affect the power of theta in behaving animals, even though the drug attenuated reticular stimulation-evoked and carbachol-induced power of theta in anesthetized animals. The lack of effect in behaving animals was regardless of the strength of theta power. NBQX was not only ineffective in the background of high power during the first 5 min of novel environment exploration but also during the later period of exploration when theta power was reduced to about 50% of that observed in the first 5 min. Similarly, NBQX failed to attenuate theta power in the formalin test, where it is about half of that observed during animal ambulation in a familiar environment.

In addition to its effect on hippocampal theta activity in anesthetized animals, AMPAR also influenced septal-mediated modulation of the CA1 pyramidal cell excitability evoked by afferent stimulation. Microinjection of NBQX in the MS attenuated the suppression of CA1 PS evoked by intraseptal carbachol. However, the effect of NBQX is relatively weak as the effect on the suppression is only seen using a 75% PS where the strength of carbachol-induced inhibition is reduced as compared to 25% PS. The same lack of effect was also observed with the suppression of 75% PS that was evoked with RPO stimulation at T V, which is a supra-maximal intensity for eliciting suppression (Jiang and Khanna, 2004). Together with the effect of NBQX on theta frequency and power, these findings suggest that intraseptal glutamate is involved in a spectrum of MS-mediated network responses.

Interestingly, the effect of intra-MS NBQX on nociceptive behaviors varied with the length of delay between the treatment and formalin injection. It was observed that NBQX had a stronger effect on nociceptive behavior when it was microinjected closer to the time of formalin injection. The greater behavioral efficacy with 5 min pre-treatment time likely reflects a greater septal availability of the antagonist over the time course of glutamate accumulation in MS, as shown by the amperometric analysis in the present study. Indeed, a gradual accumulation of extracellular glutamate was observed reaching a peak around 40th min after hind paw injection of formalin. The gradual nature of accumulation of glutamate and its dissipation suggests that the accumulation does not measure moment-to-moment changes in glutamatergic transmission in the formalin test.

Interestingly, both the carbachol- and RPO-induced suppression is mediated by septal cholinergic neurons (Zheng and Khanna, 2001). Furthermore, the septal cholinergic neurons are also implicated in nociception (Khanna and Sinclair, 1992; Zheng and Khanna, 2001; Aloisi et al., 2002; Jiang et al., 2018). These neurons, however, play a low-key role in theta generation in awake animal and sensorimotor behaviors (Leung, 1984; Bland, 1986; Buzsáki, 2002; Vandecasteele et al., 2014; Tsanov, 2017). This, then, raises the possibility that cholinergic neurons are a common target for septal AMPAR mediated changes in pyramidal cell excitability and modulation of nociception. Indeed, a relatively recent *in vitro* study also suggests that glutamatergic transmission at AMPAR can excite cholinergic neurons (Robinson et al., 2016). Modulation of frequency may involve the modulation of septal GABAergic and glutamatergic neurons, both of which are important in theta generation (Bender et al., 2015; Fuhrmann et al., 2015).

Besides AMPAR, the septal glutamate NMDAR also affects theta and behavior. For example, the glutamate NMDA receptors have been implicated in sensorimotor behavior and power of theta (Bland and Oddie, 2001; Bland, 2009; Ma et al., 2012; Ang et al., 2015; Bender et al., 2015; Robinson et al., 2016; Bortz and Grace, 2018). In the context of theta, intraseptal AP5, an antagonist at NMDAR, evoked a robust decrease in theta power under varied conditions, including on (a) exploration in a familiar environment (Leung and Shen, 2004) and (b) peripheral application of aversive/noxious stimuli (Bland et al., 2007). Taken together with current findings this suggests that intraseptal glutamatergic transmission at AMPAR cooperates with septal NMDAR in the modulation of range of septal functions, including different aspects of theta activation and septal modulated nociceptive and sensorimotor behaviors.

In conclusion, the present study shows that intraseptal glutamatergic transmission at AMPAR modulates a spectrum of physiological functions mediated by MS, including the generation of theta wave activity to novelty. However, the glutamatergic inputs that may be involved remain unclear. The MS is rich in glutamatergic neuropil and receives glutamatergic from a variety of CNS regions, including the supramammillary nucleus (Leranth and Kiss, 1996; Hajszan et al., 2004; Manseau et al., 2005; Fuhrmann et al., 2015; Ruan et al., 2017). Interestingly, the supramammillary nucleus not only modulates septo-hippocampal theta activation, including theta activity induced by noxious stimuli, but also influences arousal, sensorimotor behavior, and the regional neurons are excited on exposure to a novel environment (Wirtshafter et al., 1998; Pan and McNaughton, 2004; Kocsis and Kaminski, 2006; Ma and Leung, 2007; Ito et al., 2009; Ariffin et al., 2010; Pedersen et al., 2017). Furthermore, the septal glutamatergic neurons increase their activity during arousal (Fuhrmann et al., 2015; An et al., 2021). They may also contribute to the local glutamatergic transmission.

## Data Availability Statement

The original contributions presented in the study are included in the article/Supplementary Material, further inquiries can be directed to the corresponding author.

## Ethics Statement

The animal study was reviewed and approved by Institutional Animal Care and Use Committee (IACUC) National University of Singapore (NUS).

## Author Contributions

KI conducted all experiments and analyzed the data. KI and MA build the figures. KI and SK interpreted the data. KI, MA, and SK wrote the manuscript. All authors contributed to the article and approved the submitted version.

## Conflict of Interest

The authors declare that the research was conducted in the absence of any commercial or financial relationships that could be construed as a potential conflict of interest.
