# Supplementary material for: Modulation of Septo-Hippocampal Neural Responses in Anesthetized and Behaving Rats by Septal AMPA Receptor Mechanisms
Source: Front Neural Circuits. 2021 Jun 4;15:663633. doi: 10.3389/fncir.2021.663633 (PMC8220821; doi:10.3389/fncir.2021.663633)
Supplement: Supplementary file 1 [file Data_Sheet_1.docx]

Supplementary Material

## Supplementary Figure 1


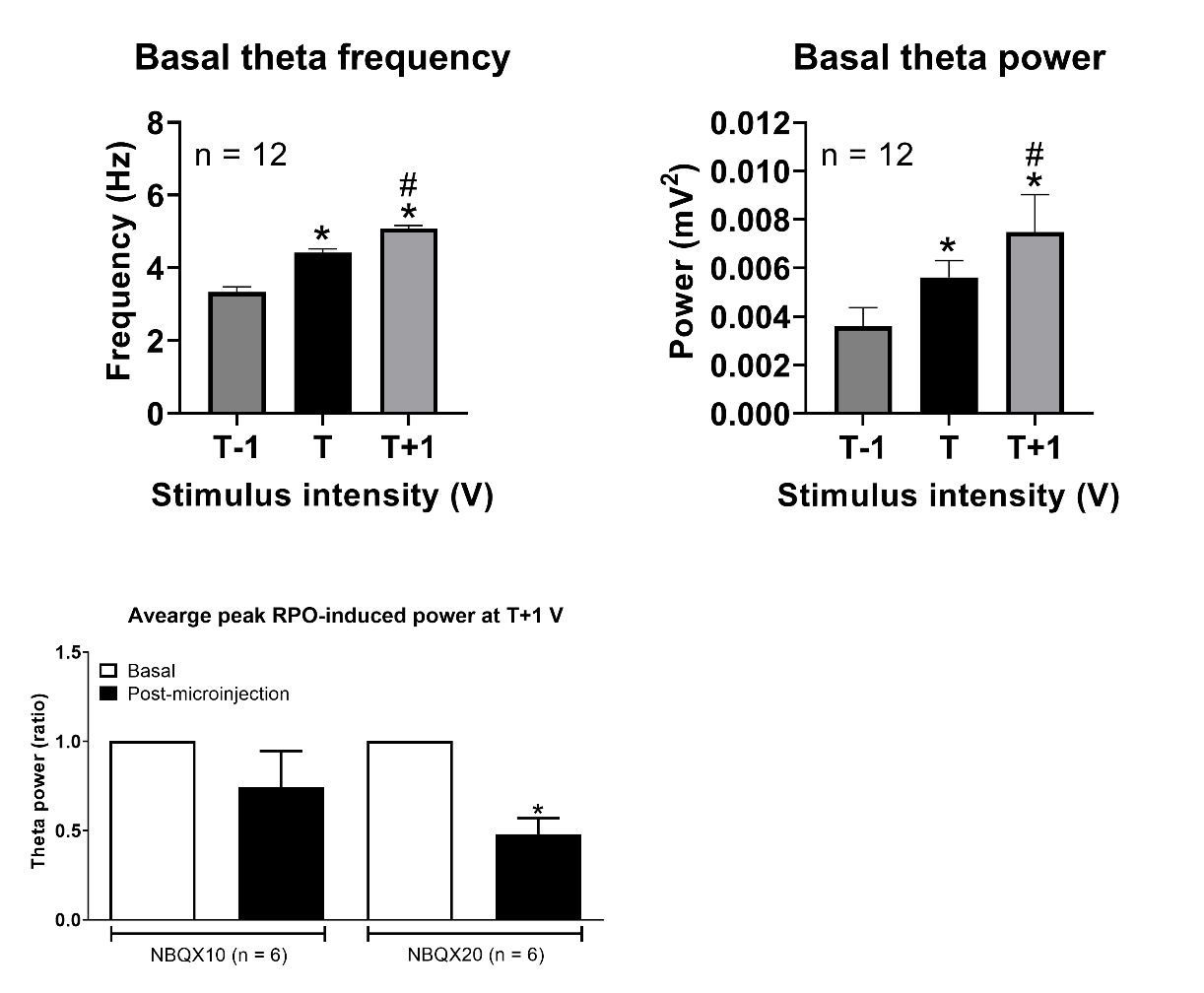


**(A)**

**(B)**

**(C)**

**Supplementary Figure 1.** **NBQX antagonizes theta peak power evoked on a high intensity RPO stimulation.**

The top panels illustrate the effect of RPO stimulation at different intensities on hippocampal theta frequency **(A)** and power **(B)** in absence of any drug treatment (Basal responses). The stimulus intensity that evoked threshold theta activity of $\sim$ 4-5 Hz is labeled as T V. Both RPO-evoked theta frequency and power were significantly higher on RPO stimulation at stimulated at T+1 V. **(C)** Histogram showing FFT theta peak power at T+1 V before and after microinjection of different doses NBQX in separate experiments. Data are mean ± S.E.M. Significant difference (p < 0.05): **(A-B)** * vs. T-1, # vs. T, (**C)** * NBQX20 vs corresponding Basal response; one-way RM ANOVA followed by Newman-Keuls post-hoc test.

## Supplementary Figure 2


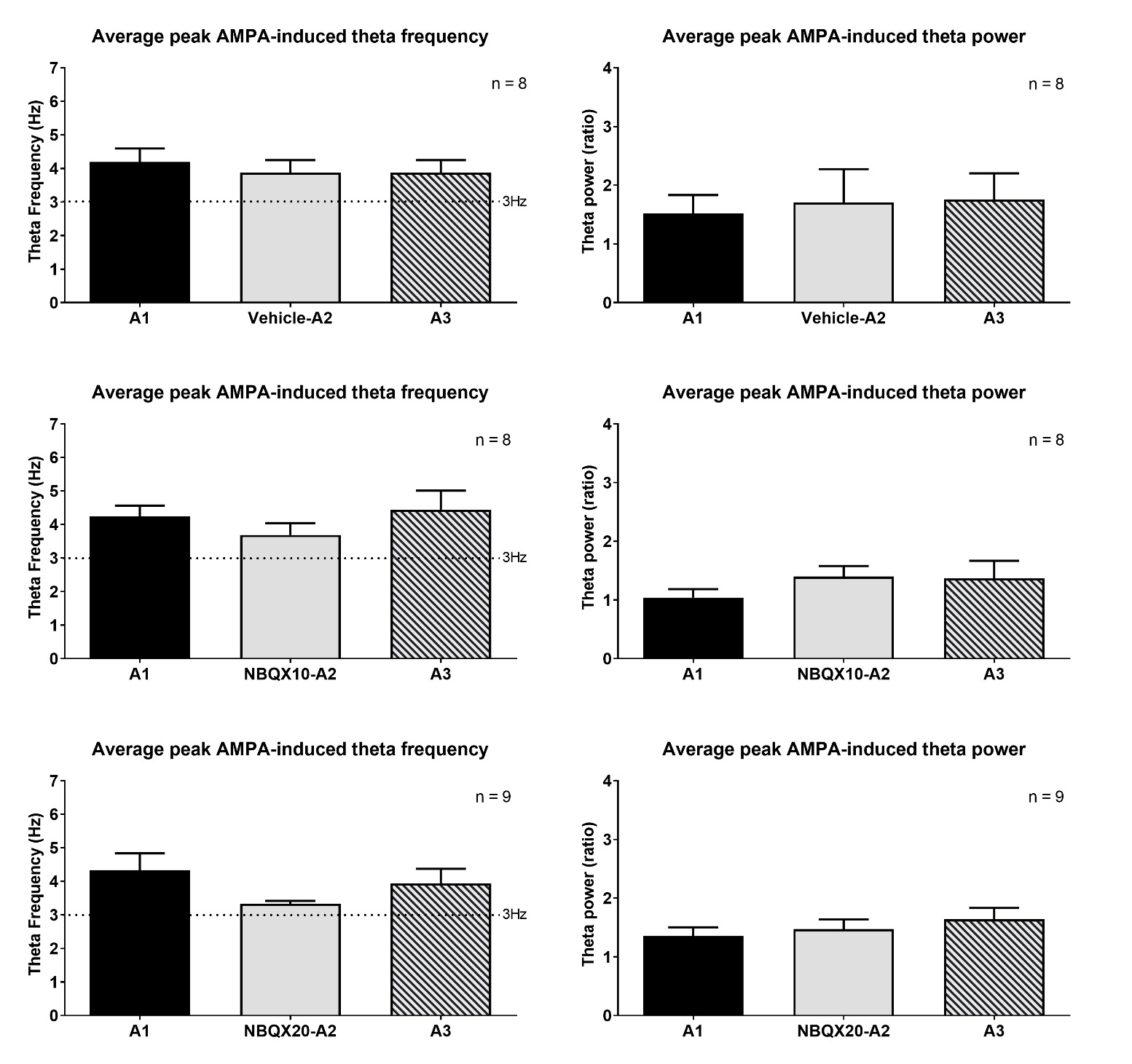


**(A)**

**(B)**

**(C)**

**(D)**

**(E)**

**(F)**

**Supplementary Figure 2.** **The frequency and power of residual theta observed on microinjection of AMPA in NBQX pretreated animals**.

Histograms illustrates the fast Fourier transform (FFT; resolution of 0.5 Hz) average peak theta frequency (Average peak AMPA-induced theta frequency; left) and power (Average peak AMPA-induced theta power; right) following miroinjection of AMPA in animals pretreated with either vehicle or one of the two concentrations of NBQX before A2. The nomenclature used in the figure is as in Figure 3. The average peak values were the average of the highest values in 2 contiguous minutes within the first 5 min following AMPA microinjection. The power of theta activity was expressed as a ratio of the power of spontaneously occurring theta activity. Data are mean ± S.E.M.

## Supplementary Figure 3

**
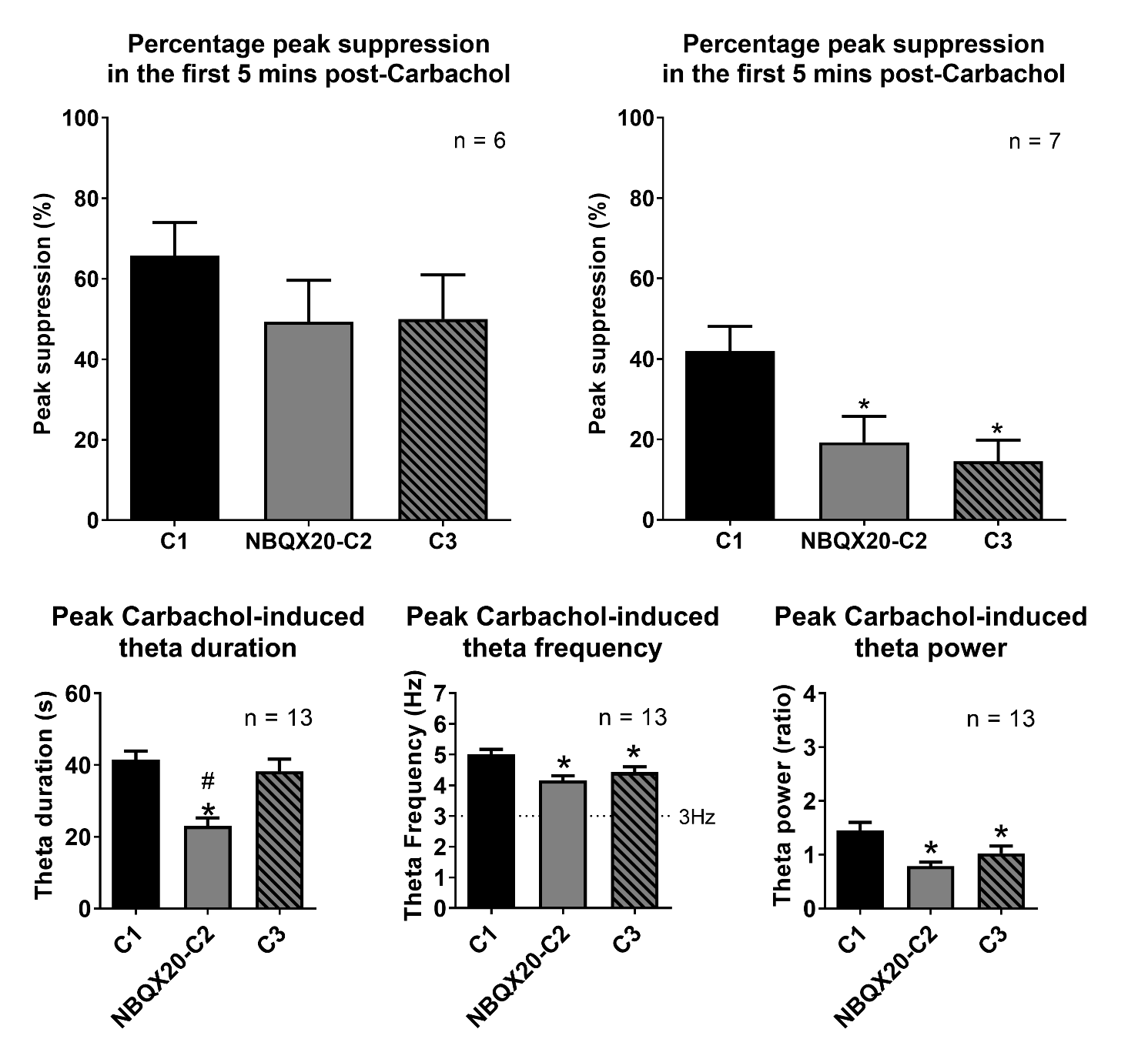
**

**(B)**

**(A)**

**(C)**

**(E)**

**(D)**

**Supplementary Figure 3.** **The AMPA receptor antagonist, NBQX, antagonizes carbachol-induced suppression of CA1 population spike and the concomitant theta activation.**

The experimental protocol is as described in Figure 3, except that carbachol (0.156 μg/μl, 0.5 μl) was injected into the MS (labeled C1, C2, and C3). NBQX (20 μg/μl) was administered 15 min before C2, thus labeled NBQX20-C2. Carbachol-induced responses were continuously monitored for 10 min. In separate group of animals, effects of NBQX were tested against CA1 PS evoked at either 25% **(A)** or 75% **(B)** of maximal amplitude. NBQX effects on PS amplitude was quantified as the percentage peak suppression induced in the first 5 min following carbachol microinjection, calculated as described in Figure 3. NBQX pre-treatment decreases the percentage peak suppression of the 75% PS **(B)**. The changes in theta wave activity were similar across the two groups of animals and, therefore, combined **(C-E)**. The duration of theta was calculated as the sum of visible theta in 1-min blocks. The peak Carbachol-induced theta duration **(C)** represents the average of highest values of theta duration in 2 contiguous minutes in the first 5 min following microinjection of carbachol. Histogram **(D** and **E)** illustrates the fast Fourier transform (FFT; resolution of 0.5 Hz) parameters of theta wave activity monitored in **(C)**. Data are mean ± S.E.M. Significant difference (p < 0.05): * vs. C1, # vs. C3; one-way RM ANOVA followed by Newman-Keuls post-hoc test.

## Supplementary Figure 4


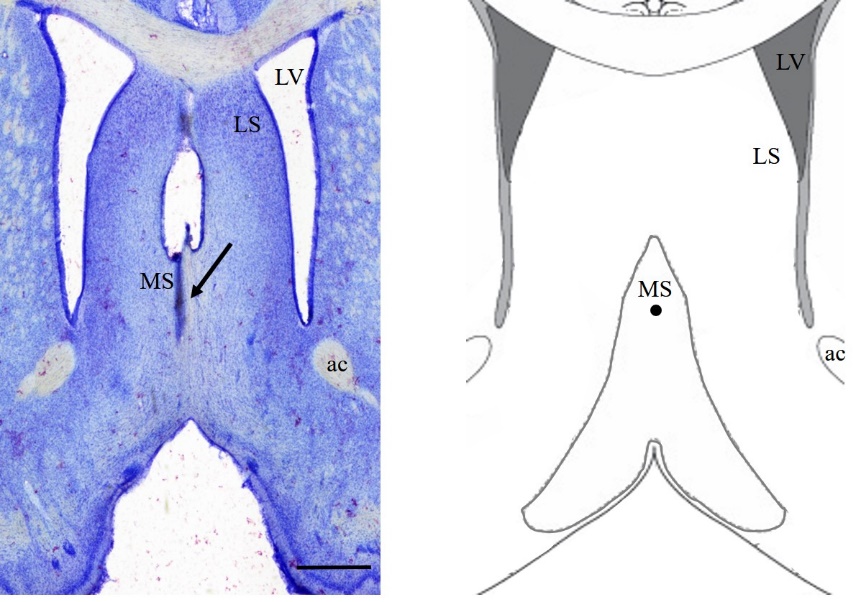

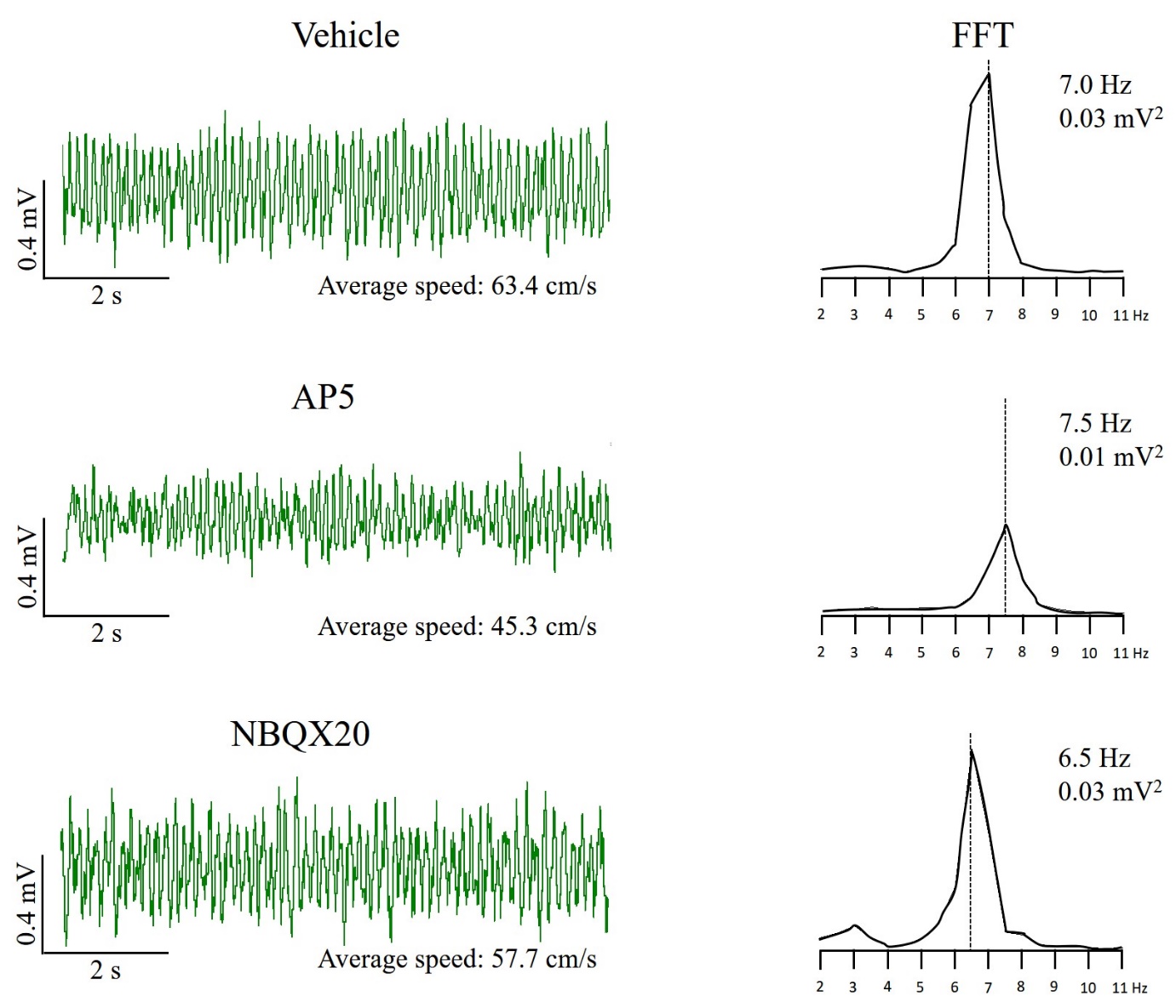


**(B)**

**(A)**


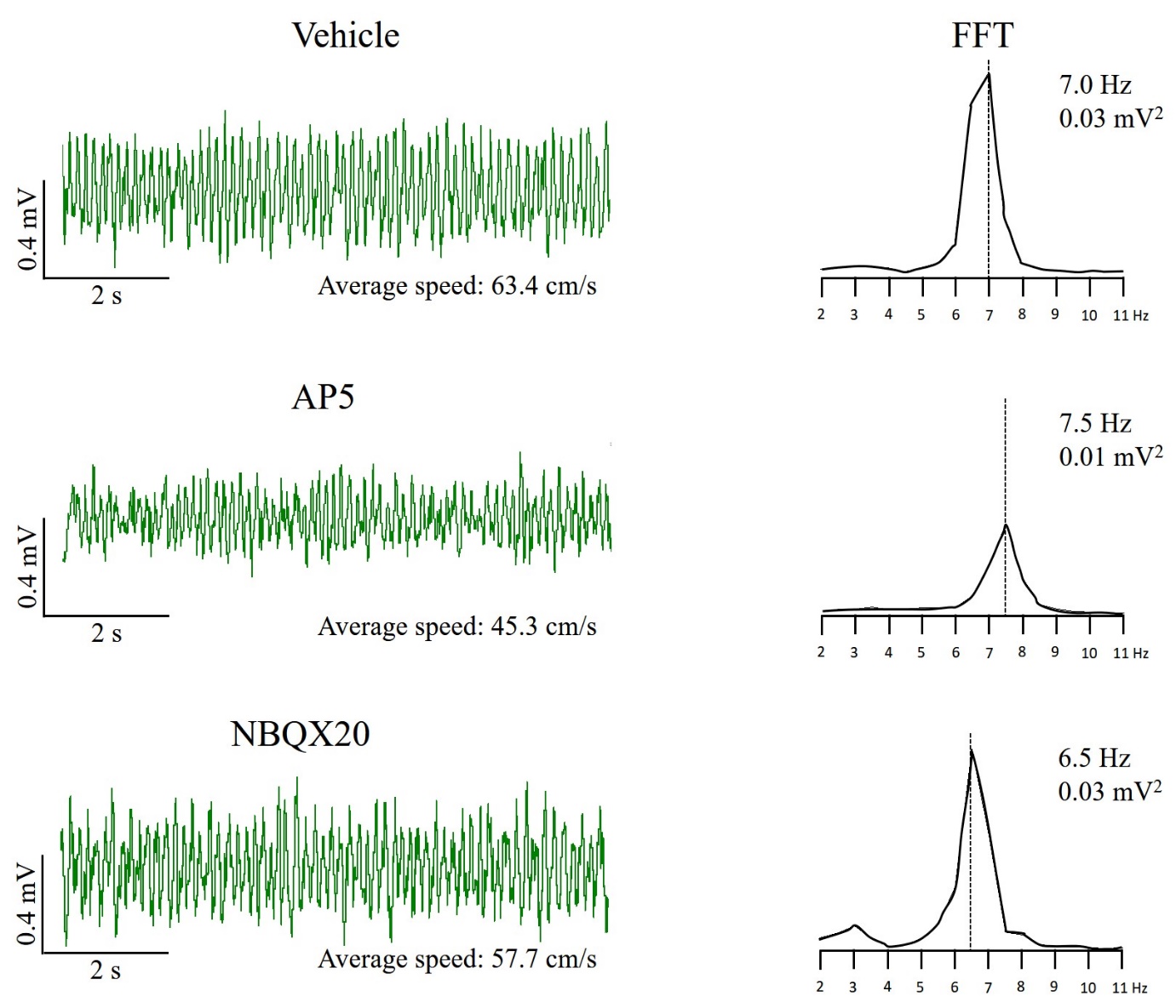


**Supplementary Figure 4.** **Intraseptal microinjection of NBQX affects theta activation during exploration in novel open field.**

**(A)** Digital representative image of the microinjection site in the MS. The arrow (left) points to the Alcian blue dye spot which reflects the microinjection site, marked by the black dot (right) in the diagrammatic representation. The scale bar on the image represents 1.0 mm. The diagram on right is adapted from Paxinos and Watson (2014). **(B)** Representative waveforms of the hippocampal field activity recorded from the stratum radiatum of the hippocampus. The waveforms are taken from animals that received intraseptal vehicle microinjection (0.5 μl, top trace; ‘Vehicle’) or NBQX (20 μg/μl, 0.5 μl, lower trace; ‘NBQX20’). The waveforms represent the first 10 s of field activity following the placement of the animals into the open field chamber. The FFT (frequency resolution of 0.5 Hz) of field activity is shown to the right of each trace. The FFT theta peak frequency and FFT theta peak power are shown on top and right of each FFT. The average speed of ambulation in the 10 s period is indicated below each trace. A clear theta wave activity was observed across all groups. ac, anterior commissure; LS, lateral septum; LV, lateral ventricle; MS, medial septum.

## Supplementary Figure 5

**(A)**


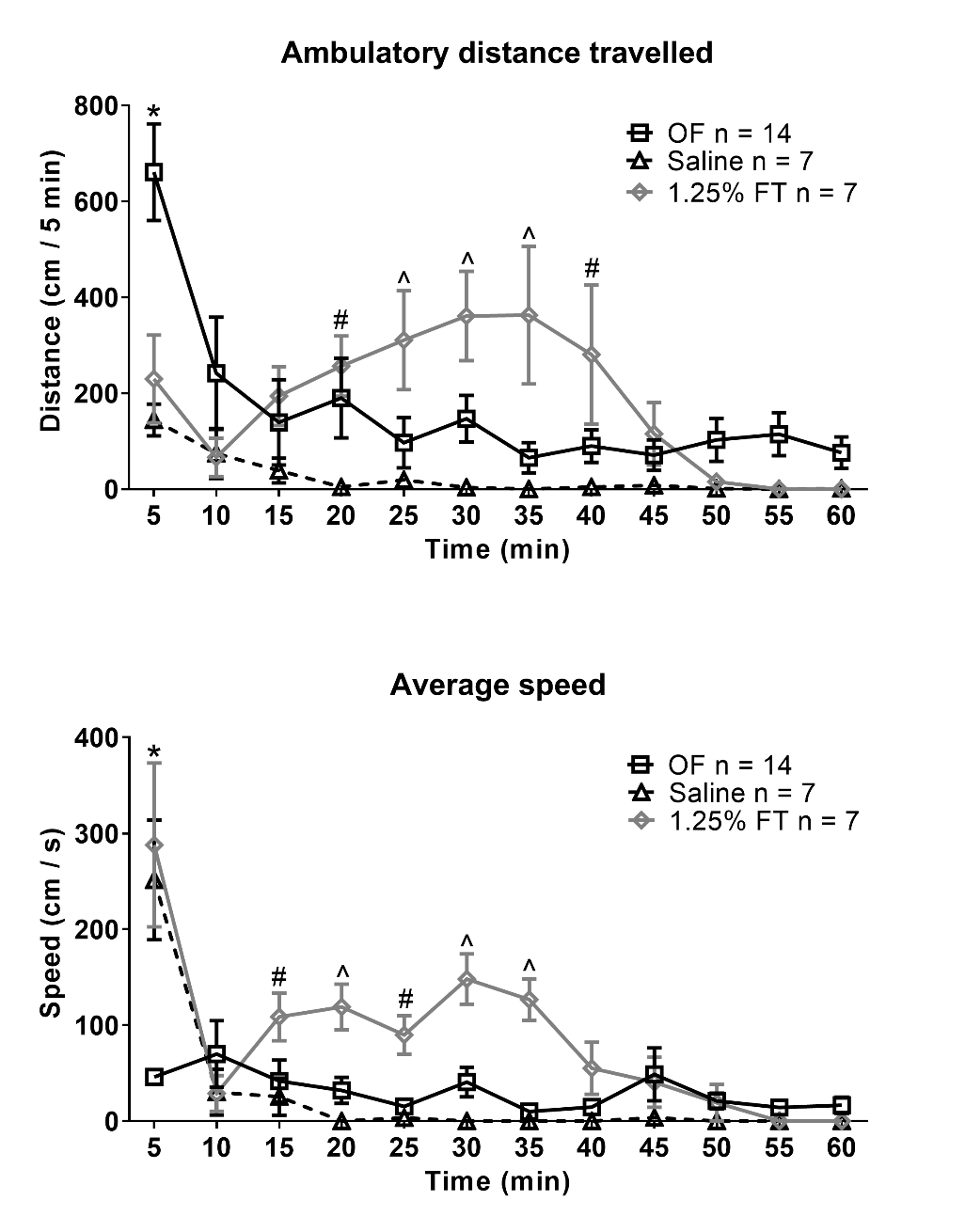


**(B)**

**Supplementary Figure 5.** **Ambulatory distance and speed of animals during amperometric recording.**

The ambulatory distance (**A**) and speed (**B**) were monitored simultaneously with amperometric signals as described in Figure 6. The pattern of change in open field (OF) and the formalin test (1.25% FT) is as generally seen with the two models. Hind paw injection of saline (‘Saline’ on the plot) evoke a brief increase in ambulation early on after injection. Data are mean ± S.E.M. Significant difference (p < 0.05): * OF vs saline and 1.25%FT, # saline vs. 1.25% FT, ^ 1.25% FT vs OF and saline; two-way RM ANOVA followed by Bonferroni post-hoc test.
